# Invasion and metastasis in cancer: molecular insights and therapeutic targets

**DOI:** 10.1038/s41392-025-02148-4

**Published:** 2025-02-21

**Authors:** Yongxing Li, Fengshuo Liu, Qingjin Cai, Lijun Deng, Qin Ouyang, Xiang H.-F. Zhang, Ji Zheng

**Affiliations:** 1 Department of Urology, Urologic Surgery Center, Xinqiao Hospital, Third Military Medical University (Army Medical University), Chongqing, China; 2 State Key Laboratory of Trauma and Chemical Poisoning, Third Military Medical University (Army Medical University), Chongqing, China; 3 Lester and Sue Smith Breast Center, Baylor College of Medicine, Houston, TX USA; 4 Dan L. Duncan Cancer Center, Baylor College of Medicine, Houston, TX USA; 5 Department of Molecular and Cellular Biology, Baylor College of Medicine, Houston, TX USA; 6 McNair Medical Institute, Baylor College of Medicine, Houston, TX USA; 7 Graduate School of Biomedical Science, Cancer and Cell Biology Program, Baylor College of Medicine, Houston, TX USA; 8 Department of Medicinal Chemistry, Third Military Medical University (Army Medical University), Chongqing, China

**Keywords:** Metastasis, Cancer therapy, Cancer

## Abstract

The progression of malignant tumors leads to the development of secondary tumors in various organs, including bones, the brain, liver, and lungs. This metastatic process severely impacts the prognosis of patients, significantly affecting their quality of life and survival rates. Research efforts have consistently focused on the intricate mechanisms underlying this process and the corresponding clinical management strategies. Consequently, a comprehensive understanding of the biological foundations of tumor metastasis, identification of pivotal signaling pathways, and systematic evaluation of existing and emerging therapeutic strategies are paramount to enhancing the overall diagnostic and treatment capabilities for metastatic tumors. However, current research is primarily focused on metastasis within specific cancer types, leaving significant gaps in our understanding of the complex metastatic cascade, organ-specific tropism mechanisms, and the development of targeted treatments. In this study, we examine the sequential processes of tumor metastasis, elucidate the underlying mechanisms driving organ-tropic metastasis, and systematically analyze therapeutic strategies for metastatic tumors, including those tailored to specific organ involvement. Subsequently, we synthesize the most recent advances in emerging therapeutic technologies for tumor metastasis and analyze the challenges and opportunities encountered in clinical research pertaining to bone metastasis. Our objective is to offer insights that can inform future research and clinical practice in this crucial field.

## Introduction

**Fig. 1:**
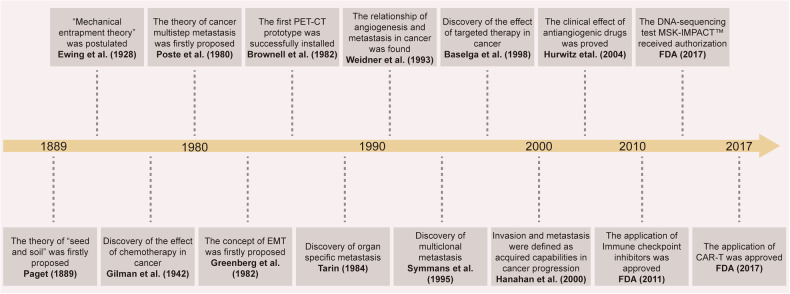
Historical progression in cancer metastasis research: from the discovery of important theoretical mechanisms to the application of clinical drugs. FDA food and drug administration

Tumor metastasis represents a pivotal event in the progression of malignancy, accounting for over 90% of cancer-related deaths and posing a formidable challenge to the clinical management of the vast majority of advanced patients with cancer.^1–3^ This intricate process encompasses the uncontrolled proliferation of primary tumor foci and the transmigration of cancerous cells across tissue barriers, which contributes to new lesions in distant organs. This substantially compromises patients’ survival rates and quality of life.^4–6^ The polymorphism and complexity of tumor metastasis are evident in its impact on virtually all vital organs throughout the body, including the lungs, liver, brain, and bones. The intricate interplay between cancer cells and the microenvironment of the target organ represents the core of this metastatic cascade. This interplay involves dynamic changes in numerous cytokines, growth factors, and signaling pathways, collectively creating a microenvironment conducive to tumor growth and dissemination^7–9^ (Fig. 1).

While significant advancements have been made in fundamental research on tumor metastasis, effectively translating these findings into clinical practice remains a considerable challenge. Current clinical studies usually prioritize the development and evaluation of pharmacological treatments, with a relative lack of emphasis on the comprehensive understanding of metastasis mechanisms, the specific mechanisms underlying organ-specific metastasis, and the exploration of targeted therapies. Therefore, this review aims to examine the multidimensional nature of tumor metastasis, mainly focusing on bone, brain, liver, and lung metastasis as archetypal representatives. By integrating the “seed and soil” theory with the “multiclonal metastasis” theory, we aim to analyze the interactions between tumor cells and the microenvironments of various organs, thereby uncovering the pivotal signaling pathways and regulatory mechanisms underlying metastasis. Moreover, an exhaustive review of existing clinical research and trials will be conducted to evaluate the efficacy of pharmacological, non-pharmacological, and comprehensive management strategies in treating tumor metastasis. The objective of this endeavor is to provide a more comprehensive and scientific basis for the clinical management of tumor metastasis. We aim to identify the key challenges within the field and propose forward-thinking solutions, with the ultimate goal of fostering the continuous optimization and advancement of diagnostic and therapeutic strategies for tumor metastasis.

## Clinical significance of cancer metastasis

**Fig. 2:**
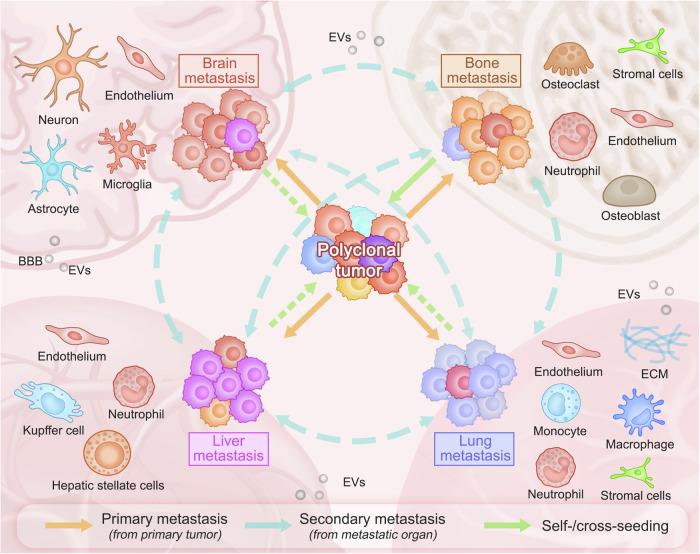
Metastasis of cancer cells. Tumor cells with inherent genomic instability accumulate mutations leading to significant heterogeneity. Metastasis involves the colonization of distant sites by various clones from the primary tumor, resulting in polyclonal metastasis. Studies on various solid cancer metastasis patterns support this concept by revealing polyclonal seeding and heterogeneity within metastatic lesions. The bidirectional flow of cancer cells, as proposed by tumor self-/cross- seeding (indicated by green and orange arrows) or secondary metastasis from metastatic site (blue arrows), adds metastasis complexity, indicating potential intra- and interpatient heterogeneity in treatment response and resistance

Metastasis represents a defining characteristic of malignancy, with a documented causal role in over 90% of cancer-related deaths.^10^ The brain, lungs, liver, and bones are the most common sites for metastasis, with various cancer types exhibiting distinct patterns of dissemination to specific organs or tissues^11^ (Table 1). This organ affinity indicates that metastasis is driven by intricate biological mechanisms rather than mere statistical correlation.^12^ A comprehensive understanding of the epidemiology of cancer metastasis is essential for identifying high-risk populations and the development of targeted screening programs. Recognizing organ-specific tendencies in different cancers facilitates more effective monitoring and management of patients by clinicians. This knowledge is crucial for improving patient outcomes and reducing the global burden of cancer-related mortality (Fig. 2).

**Table 1:** The tropism of cancer metastasis

Type of metastasis	Annual incidence (per 100,000 individuals)	Incidence in patients with cancer (%)	Primary cancer site										References
			Breast	Lung and bronchus	Pancreas	Prostate	Liver	Colorectum	Intestines	Stomach	Bones and joints	Melanoma	
Bone	18.8	5.1	+++	++++	+	+++	+	+	/	+	/	+	^607^
Brain	8.3–10.3	1.9–9.6	+++	++++	/	/	/	/	/	/	/	+	^13,15–19,608^
Liver	6.4	5.14–6.46	+	++	++++	+	+	+++	+++	+++	/	+	^38,609^
Lung	4	17.92	+	++++	+++	+	++	++	/	++	++++	+	^42^

Extremely High frequency: ++++; high frequency: +++; medium frequency: ++; low frequency: +

Approximately half of all intracranial tumors are brain metastasis. The most common site of intracranial metastasis is the brain parenchyma. In addition, cancer can metastasize to the skull, dura mater, and meninges, with metastasis occurring simultaneously, which can result in severe neurological complications.^13^ As evidenced by published studies, the incidence of brain metastasis ranges from 8.3 to 14.3 per 100,000 individuals,^14–16^ with a prevalence of 1.9% to 9.6% among patients with cancer.^17–19^ Previously, the diagnosis of brain metastasis primarily depended on the pathological verification of surgically removed specimens and autopsies of deceased patients. However, considering that neurosurgeons usually treat patients with localized brain metastasis and those with longer expected survival rates,^20^ and not all autopsies include central nervous system examinations, the incidence of brain metastasis has probably been underestimated.^13,17^ The efficacy of chemotherapy in extending survival periods^21–23^ has increased the likelihood of cancer cells spreading to the brain. Furthermore, the ongoing advancement in imaging technologies has improved detection, contributing to the increased incidence of brain metastasis.^13,21^

Statistical data indicates that over 19 million new cancer cases are registered worldwide annually, with over 60% of these cases ultimately developing into metastatic disease.^24,25^ Bone metastasis represents a substantial proportion of these cases. It is noteworthy that the incidence of bone metastasis in patients with breast, prostate, and lung cancers is as high as 75, 70–85, and 40%, respectively.^26–29^ Bone metastasis affects bone health and often results in severe complications, including skeletal-related events (SREs), such as fractures and increased pain. These complications have a markedly deleterious impact on patients’ quality of life and a considerable increase in the overall medical burden.^30,31^ In patients with prostate cancer, the three-year and five-year survival rates are 50 and 65%, respectively, in patients with bone metastasis compared to those without, which demonstrates the adverse impact of bone metastasis on the survival of patients with cancer.^32^ Furthermore, in patients with lung cancer and bone metastasis, the incidence of SREs within one year of diagnosis is as high as 55%, resulting in a notable reduction in survival rates.^33^

Liver metastasis is a prevalent complication in the advanced stages of various cancers, affecting approximately 5% of patients with cancer. It is notably prevalent in young women with breast cancer and young men with colorectal cancer.^34^ However, with increasing age, the types of primary cancers causing liver metastasis to diversify, extending beyond the lung, pancreatic, and colorectal cancers to include esophageal, gastric, and small-intestine cancers.^35–38^ The liver’s distinctive physiological structure and function render it a “haven” for numerous tumor cells,^10,39^ contributing to elevated liver metastasis rates in countries like the United States compared to those of primary liver cancer.^40,41^ Notably, the survival rate of patients with liver metastasis is markedly inferior, with a one-year survival rate of only 15.1%, which is considerably lower than the 24.0% observed in patients without liver metastasis.^34^ Moreover, the consumption of medical resources is exacerbated by liver metastasis, thereby imposing a significant economic and psychological burden on the patients’ families and society.

The incidence of lung metastasis is as high as 17.92 per 100,000 individuals^42^ and commonly occurs in cancers such as lung and colorectal cancers.^43–45^ Approximately 4% of patients with cancer present with synchronous lung metastasis at the time of diagnosis.^42^ Among patients with primary lung cancer, the proportion of patients with lung metastasis was as high as 13%. In contrast, it was the lowest in patients with prostate cancer, at only 0.5%, with this rate continuously increasing.^42^ This phenomenon may be closely related to the widespread use of advanced imaging technologies, such as CT and PET, which facilitate more precise detection of lung metastasis.^46^ However, the prognosis for patients with lung metastasis is generally poor, with overall survival rates significantly lower than those of patients without lung metastasis. This cohort predominantly comprised elderly males with late-stage cancers^42^]. Therefore, improving early screening, accurate diagnosis, and comprehensive treatment of this high-risk population is imperative.

## Mechanisms of cancer metastasis

### Organ tropism and metastasis theories

Metastasis is a defining characteristic of malignancy that presents significant challenges in oncology, owing to the spread of cancer cells from the primary sites to distant organs. Metastatic cells often exhibit organ-specific preferences, known as “organ tropism”. Determining this predilection is vital for advancing preventive and therapeutic measures. Two pivotal theories, the “seed and soil” hypothesis, and the “multiclonal metastasis” theory, enhance our understanding of bone tropism. Introduced by Paget in 1889, the “seed and soil” hypothesis posits that metastasis is not random.^47^ It proposes that the “seed” (cancer cells) requires a conducive “soil” (metastatic site) for successful growth, with specific tissue niches providing factors that facilitate their development. Furthermore, the origin of metastatic cells is not limited to the action of a singular dominant “seed.” Instead, it is the collective contribution of various cancer cell subpopulations within the primary tumor, known as “multiclonal metastasis,” orchestrating this metastatic process.^48^ This underscores the inherent heterogeneity within primary tumors, which is crucial for their metastatic capabilities.

#### Seed and soil theory

The “seed and soil” theory offers a framework for understanding the intricate process of cancer metastasis. The successful spread of cancer cells (the “seed”) to distant organs or tissues depends on both their intrinsic properties and the distal colonized microenvironment (the “soil”). Metastasis occurs when circulating tumor cells (CTCs) interact with the microenvironment of a distant organ, creating conditions conducive to their survival, proliferation, and colonization.^49^ After detachment from the primary tumor, CTCs enter the bloodstream and must survive a hostile environment, evade immune surveillance, adhere to the narrow capillaries of distant organs, and extravasate into the surrounding tissue. This extravasation step is particularly significant for organ tropism, as it determines whether cancer cells can establish a niche within specific target organs (Fig. 2). We summarized key signaling molecules and pathways reported for organ tropism in Table 2.

**Table 2:** The molecules/signaling pathways critical for organ tropisms

Distal seeding organ	Primary cancer type	Key cellular participants	Signaling molecules/pathway/mechanisms	References
Bone	Prostate	Osteocyte	GDF15, CCL2, RANKL	^208,610^
	Breast	Osteoclast	CXCR4, RANKL	^611^
	Breast	Osteoblast	CXCL12, RANKL, PTHrP	^192,612,613^
	Prostate/breast/kidney	Osteoclast	RANK, RANKL, OPG	^614–617^
	Prostate	Osteoclast	NRP2	^618^
	Lung	Osteoclast	CXCR4, VCAM1, ADAM17	^619,620^
	Lung	Osteoclast	IL-20	^621^
	Lung/colon	Macrophage	NF-κB, STAT3/CCL5	^622,623^
	Lung	Macrophage	CSF-1	^624,625^
	Lung	ECM	MMP14	^626^
	Kidney	RCC	PLOD2/ hypoxia	^627,628^
	Colon	Bone niche	BMPs, TGF-β, WNT	^623,629^
Brain	Breast	Endothelium	MUC1, VCAM1, VLA-4	^220^
	Breast	Neurons	Glutamate	^231^
	Breast	Brain niche	Fatty acid	^630^
	Breast/lung	Blood brain barrier	Tight junction degradation	^21,218,631–633^
	Breast/lung	Vasculature	VEGF	^634–637^
	Breast	Astrocyte Pericyte	S1PR3, IL-6, TNF-α, IL-1β	^638,639^
Liver	Pancreatic	Kupffer cells, Hepatic stellate cells (HepSCs)	TGF-β, VEGF, Fibronectin	^182^
	Breast	Hepatocytes	αvβ3 integrin	^640^
	Breast	Kupffer cells, Hepatic stellate cells (HepSCs)	Claudins	^641^
	Lung	Liver sinusoidal endothelial cells	EGFR^mutated^	^53,642^
	Lung	Kupffer/Neutrophils,	E-/P-selectin,VCAM1	^643^
	Colon	Kupffer cells, Hepatic stellate cells (HepSCs)	KRAS, NRAS, BRAF, microsatellite instability	^644–647^
	Melanoma	Kupffer cells, Hepatic stellate cells (HepSCs)	Monosomy 3	^648^
Lung	Breast	Tenascin C	Notch	^251,252^
	Melanoma	Endothelium	SPARC, VCAM1	^253^
	Breast/ melanoma	Actin	CXCR4, CCR7	^254^
	Breast	Macrophage	cGAS–STING, CCL5, CCL7	^649,650^
	Breast	Neutrophil	STC1	^651^
	Breast	Monocyte	acetyl-CoA, NF-κB	^652^
	Breast	Neutrophil	Fatty acid, lipid metabolism	^95,653^
	kidney	Renal cell	PRMT2, WNT5A	^654^
	Breast/ kidney	Lung niche	EVs	^86,87,655,656^

Equally important is the “soil,” or the microenvironment at the metastatic site. This environment is composed of a complex array of growth factors, cytokines, and extracellular matrix components, and diverse cell types (Table 2). Cancer cells, tissue-specific niches, and immune cells engage in intensive cell-cell communication to shape a tumor-favoring ecosystem. Tissue structure also influences metastasis patterns; for example, the lymphatic system often serves as a primary route for dissemination, with lymph nodes providing initial sites for cancer cell trapping and proliferation before further spreading via lymphatic and circulatory systems.^50^ Likewise, the liver and lungs are common metastasis sites due to their distinctive blood flow patterns.^51,52^ These insights underscore the complex interplay between the genetic makeup of cancer cells and permissive distant microenvironments. Recognizing these “seed and soil” dynamics may guide the development of more effective therapeutic strategies that disrupt supportive niches and impede the colonization and growth of metastatic cancer cells. Continued research will refine our understanding of bone metastasis and inform improved management of various cancers.

The blood and lymphatic circulation patterns play a crucial role in determining metastatic sites. Anatomical factors greatly influence the site at which cancer cells disseminate, with the liver and lungs being common metastasis sites owing to their distinctive blood flow patterns.^51,52^ For example, gastrointestinal cancers often metastasize to the liver owing to the direct blood flow from the intestines via the portal vein system.^53^ Additionally, adhesion molecules such as integrins and selectins expressed on cancer cell surfaces enable these cells to adhere to and invade the target organs by binding to endothelial cells.^54^ Integrin-mediated organ tropism has been illustrated in various model systems. For instance, studies using melanoma and patient-derived MDA-MB-231 breast cancer cells^55^ have shown that exosomes carrying α6β1 and α6β4 preferentially direct metastases to the lungs, whereas αvβ5-bearing exosomes facilitate liver colonization. In breast cancer, exosomes carry β3 integrin and sialylated N-glycans/integrins have been implicated in promoting brain metastasis.^56^ Furthermore, αv integrin has been shown to promote bone colonization by interacting with and dysregulating osteoclast functions.^57–60^ It has been selected for potential targets in treating bone metastasis.^61^

The influence of chemotactic factors and their receptors on organ tropism is important in this process. Specific organs secrete chemokines and growth factors that attract cancer cells to express their corresponding receptors. For instance, the CXCL12/CXCR4 axis plays a crucial role in the metastasis of breast cancer to the lungs and bones by directing cancer cells to these sites.^62^

The composition of the ECM in different organs can influence the process of metastatic colonization. Specific ECM components provide a supportive niche for metastatic cancer cells, facilitating their growth and survival. ECM proteins, such as fibronectin and laminin, enhance the adhesion and invasion capabilities of cancer cells.^63^

The formation of a pre-metastatic niche (PMN) is initiated by tumor-secreted factors, preparing distant organs for cancer cells even before their arrival. Exosomes, cytokines, and other molecular components secreted by the primary tumor can modify the microenvironment of the target organs to support metastasis. Another critical factor in cancer metastasis is immune evasion, whereby cancer cells avoid detection and destruction by the immune system to colonize new sites.^64^ For instance, some organs, such as the brain, provide a distinctive immune milieu that safeguards metastatic cells from immune surveillance.^65^ Finally, organ-specific growth factors support the growth of metastatic cells. Specific organs produce growth factors that favor the proliferation of specific types of cancer. For example, IGF-1 in bone marrow supports prostate cancer metastasis.^66^

#### Multiclonal metastasis

The “multiclonal metastasis” theory emphasizes that metastasis arises through a complex, dynamic evolutionary process (Fig. 2). Due to inherent genomic instability, tumor cells accumulate numerous mutations, resulting in substantial heterogeneity and enabling different tissues to be colonized by multiple, genetically distinct tumor clones (i.e., polyclonal metastasis).^67^ Whole-genome sequencing studies have shown that metastases can originate from intermingling multiple tumor clones across metastatic sites, highlighting the multifaceted nature of metastatic dissemination.^68^ This concept aligns with the evolutionary dynamics described by Turajlic and Swanton,^69^ who demonstrated that emerging metastatic subclones contribute significantly to genetic diversity within metastatic tumors.

Further evidence for polyclonal metastasis includes polyclonal lymph node metastases in colorectal cancer arising from disparate regions of the primary tumor,^70^ as well as breast cancer metastases driven by collective dissemination of keratin 14-expressing tumor cell clusters.^71^ In the TRACERx study, both polyclonal and monoclonal metastases were observed in non-small-cell lung cancer (NSCLC), illustrating that metastatic clones often represent expansions of subclones from the primary tumor.^72,73^ Colorectal cancer (CRC) patients, in particular, frequently exhibit polyclonal metastasis in lymph nodes compared to other organs,^74–76^ and triple-negative breast cancer (TNBC) metastasis often displays heterogeneous subclone populations characteristic of polyclonal seeding.^77^ Similar patterns have been noted in colorectal and pancreatic cancers, where distinct subclones give rise to metastatic lesions, further reinforcing the polyclonal nature of metastasis and its implications for treatment heterogeneity.^78–80^ The concept of tumor self-/cross-seeding introduces the possibility that circulating tumor cells can repopulate both primary and metastatic lesions, augmenting tumor heterogeneity even further,^81^ a phenomenon also supported by recent liver cancer studies employing novel labeling systems (Fig. 2).^82^

Collectively, these insights underscore that multiclonal metastasis has profound implications for bone metastasis and beyond. Distinct subclones may respond differently to therapeutic interventions based on their unique genetic makeup, contributing to variable treatment responses and resistance within a single patient. Understanding this complex clonal architecture is critical for developing targeted, practical strategies to manage bone metastases and improve patient outcomes.

### Metastatic cascade

**Fig. 3:**
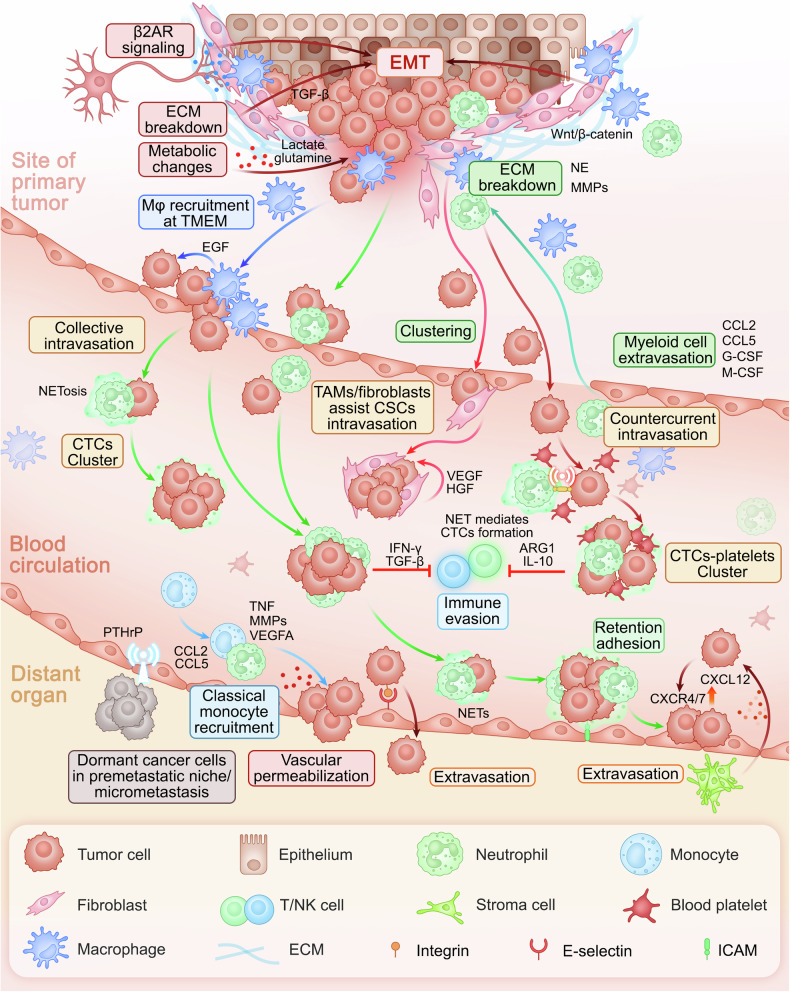
Mechanisms of the cancer metastasis cascade. At primary tumor sites, cancer-associated fibroblasts (CAFs) and stromal cells create a metastasis-conducive niche to support cancer cell EMT process, dissemination, and migration. As migrating cancer cells interact with circulating CAFs and myeloid cells to enhance survival and invasiveness while evading immune detection, they eventually reach the metastatic sites where they transition from dormancy and interact with the microenvironment to initiate active proliferation. The metastatic cascade initiated by primary tumor cells invading adjacent tissues via EMT is facilitated by CAFs that promote motility and ECM degradation. Moreover, macrophages and tumor-associated neutrophils (TANs) significantly contribute to ECM breakdown, facilitating cancer cell intravasation and survival in circulation by forming aggregates with platelets and myeloid cells to evade immune surveillance. Key interactions between cancer cells and the endothelium facilitate adhesion and extravasation into bone marrow, supported by the metabolic reprogramming of osteoblasts and osteoclasts. In addition, myeloid cells enhance cancer cell survival and metastasis through immune suppression, metabolic support, and ECM remodeling, including the crucial activities of neutrophils and macrophages in facilitating tumor cell adhesion, invasion, and metastatic proliferation at secondary sites

Metastasis is a biological process involving complex interactions between colonized cancer cells and metastatic microenvironment. At the primary tumor site, cancer-associated fibroblasts (CAFs), stromal cells, and other cells establish a “niche” conducive to tumor cell metastasis through remote regulatory mechanisms, providing the necessary microenvironment for tumor cell migration and facilitating their detachment from the primary site and embarking on their invasive journey. As tumor cells migrate, they evade immune surveillance and interact with circulating CAFs and myeloid cells to enhance survival and invasiveness. Upon reaching the metastatic site, tumor cells extravasate through frequent interactions with the local microenvironment (Fig. 3).

#### Pre-metastatic niche

The formation of PMN in the bone results from interactions between primary tumor cells and various distal niche cells. These interactions facilitate molecular and cellular changes in distant organs, setting the stage for metastatic seeding before clinically detectable collective and massive metastasis. The establishment of the PMN sets the basis for organ tropism. For instance, fibroblasts are crucial for establishing an environment conducive to metastatic colonization. The significance of fibroblasts in forming the metastatic niche is underscored by the essential role of periostin expression in the proliferation of early disseminated cancer stem cells at secondary sites, highlighting the critical influence of stromal niche signals.^83^ Moreover, the tumor-associated stroma, comprising fibroblasts and myofibroblasts, plays an active role in supporting tumor expansion by promoting neo-angiogenesis and the proliferation and invasion of cancer cells, thus aiding distal seeding, such as bone colonization.^84^ Studies on highly metastatic HCC cells have indicated that the secretion of exosomal miR-1247-3p activates fibroblasts and promotes lung metastasis in liver cancer.^85^ Secreted extracellular vesicles (sEVs) play critical roles in forming PMN in the lungs and in the preparation of lung and brain metastasis from various cancers.^86^ These sEVs contain a variety of molecules, including nucleic acids, signaling proteins, enzymes, lipids, and metabolites, that can influence cellular functions and communication.^87^

In the bone, the initiation of PMN has been attributed to VEGFR1-positive hematopoietic progenitor cells, which migrate to specific pre-metastatic sites and form clusters in anticipation of tumor cell arrival, suggesting the role of bone marrow in PMN initiation.^88^ The interaction between cancer cells and hematopoietic and mesenchymal stem/progenitor cells residing within the bone metastatic niche facilitates reciprocal communication between tumor cells and the bone metastatic stroma.^89^ In myelomas, osteoblasts undergo metabolic reprogramming in response to the primary tumor, characterized by increased glucose uptake and enhanced glycolysis. This metabolic shift facilitates the production of lactate and other metabolites that are utilized by cancer cells for energy production. Osteoclasts adopt a high-energy state, which increases bone resorption.^90^

Myeloid cells and their progenitors within PMN help establish chronic inflammation in secondary organs, which may be an immune response to infection.^91^ This inflammation, in turn, compromises the immune system’s ability to initiate an effective response, thereby facilitating the successful establishment of metastatic lesions.^92^ Primary breast tumors can induce the mobilization of CD11b+ myeloid cells to the lungs, creating an immunosuppressive microenvironment that dampens the cytotoxic activities of NK^93^ and T cells,^94^ thus promoting metastatic colonization in the lung. Furthermore, lipid metabolites in lung-resident neutrophils have been identified as significant energy sources influencing lung metastasis in breast cancer (BC).^95^ Moreover, primary lung and BC tumor growth can remotely disrupt myelopoiesis through sEVs, leading to abnormal myeloid lineage differentiation in the bone marrow, which accumulates myeloid cells in the bone marrow and supports tumor progression^96,97^ (Fig. 3). In colorectal cancer (CRC), Kupffer cells can phagocytose exosomes carrying highly expressed miR-135a-5p from the bloodstream into the liver, thereby establishing liver tropism.^98^ In addition, secreted molecules such as tissue inhibitors of metalloproteinases (TIMP-1),^99^ VEGFA,^100–102^, and CCL15^103^ can accumulate various myeloid cells in the liver and form PMN in the liver.

#### Cancer cell dissemination and intravasation

The metastatic cascade begins with the invasion of primary tumor cells into adjacent tissues. This invasive process often involves epithelial-mesenchymal transition (EMT), which enables cancer cells to acquire migratory and invasive properties. Simultaneously, primary tumor cells break the ECM and create pathways for dissemination. The local tumor microenvironment (TME) supports EMT and ECM breakdown, enabling intravasation into the bloodstream or lymphatic system (Fig. 3).

CAFs play multiple roles in cancer metastasis. One crucial mechanism involves the induction of EMT in tumor cells. CAFs secrete factors such as TGF-β, downregulating E-cadherin and upregulating N-cadherin and vimentin, signifying a mesenchymal phenotype.^104–106^ This transition promotes tumor cell motility and invasiveness, facilitating their escape from the primary tumor. In addition, CAFs drive metastasis by remodeling the ECM. Furthermore, they secrete matrix metalloproteinases (MMPs), which degrade ECM components and decrease cell-cell adhesion, aiding tumor cell invasion and migration.^107^ Moreover, direct interactions between CAFs and carcinoma cells influence invasion, with CAFs reorganizing collagen fibrils within the ECM, creating pathways for tumor cell progression.^108^ Additionally, primed CAFs support tumor cell invasion through metabolic crosstalk by secreting metabolites such as lactate and glutamine, which cancer cells readily utilize, fueling pathways that enhance their invasive potential.^109,110^ Moreover, these metabolic alterationsv not only promote primary tumor growth and metastasis but also foster immune evasion by increasing glycolysis (Warburg effect^111^), suppressing anti-tumor responses of NK cells,^112^ impairing macrophage pro-inflammatory stimulation,^113^ dysregulating myeloid cell function,^114,115^ limiting dendritic cell antigen presentation,^116^ and promoting regulatory T cell infiltration.^117^

In addition to CAFs, tumor-associated neutrophils (TANs) and tumor-associated macrophages (TAMs) support cancer invasion through ECM degradation via the secretion of MMPs.^118^ In addition, they secrete osteonectin, promoting tumor cells and ECM interaction.^119^ Further ECM remodeling is driven by TAM- and TAN-derived factors such as elastases, cathepsins, and proteinases-3.^120,121^ Changes in the bone microenvironment fuel the invasion process. Metabolic reprogramming, accompanied by metabolite release during osteoclast-mediated bone breakdown, generates a highly acidic environment. This acidosis activates proteases, such as cathepsin K, promoting ECM degradation, and facilitating the early steps of tumor cell dissociation and invasion.^122^

The tumor microenvironment for metastasis (TMEM) is a strong predictor of metastasis in human BC. Invadopodia formation, driven by interactions between macrophages and tumor cells, is key to tumor cell intravasation within TMEM.^123–125^ A paracrine loop involving macrophage-derived growth factors and tumor cell-produced colony-stimulating factor 1 fuels these interactions. In addition, transient vascular permeability within the TMEM facilitates tumor cell escape into circulation. Cancer stem cells (CSCs) accumulate at TMEM sites near TAMs. Their lower inherent migration ability suggests that CSCs may efficiently intravasate by exploiting macrophage-endothelium connections.^126,127^

In a polyomavirus middle T antigen-overexpressing BC model, TAMs promoted cancer cell intravasation by partly inducing angiogenesis via VEGFA secretion, thereby increasing blood vessel density.^128^ In addition, a subset of Tie2+ TAMs transdifferentiate into perivascular macrophages that promote vascular leakage and directly facilitate the intravasation of tumor cells.^129–131^ TANs also promote tumor cell intravasation but through different processes. One hypothesis suggests that migrating neutrophils create tunnels in the ECM, allowing tumor cells to disseminate into the vasculature. Furthermore, tumor cells may adhere directly to neutrophils, using them to facilitate transport through the endothelium^132,133^ (Fig. 3).

#### Cancer cell circulation

The circulatory system presents a harsh environment, presenting numerous obstacles to tumor cell dissemination. However, most of the tumor cells were Ki67+, suggesting they are in a state of active proliferation. CTCs have evolved strategies to avoid immune surveillance to survive and ultimately metastasize. A growing body of research elucidates these mechanisms, highlighting how CTCs evade detection and persist as they migrate to distant bone regions (Fig. 3).

One key mechanism involves physical cloaking within the platelet aggregates, obscuring them from immune surveillance.^134^ Both selectins and integrins facilitate this interaction. In addition to physical shielding, platelets release signaling factors that induce EMT in CTCs, promoting invasiveness, stemness, motility, and resistance to anoikis.^135^ CAFs can also accompany CTCs into circulation, aiding them in several ways. CAF-secreted MMPs degrade physical barriers, whereas growth factors, such as vascular endothelial growth factor (VEGF) and hepatocyte growth factor (HGF), support CTC survival. Moreover, CAFs facilitate the metabolic adaptability required for CTCs to withstand stress during circulation.^136,137^

Myeloid cells also support CTCs, often forming cellular aggregates with disseminated tumor cells. Initial in vitro studies have indicated that TANs promote aggregation of both breast and CRC cells. Subsequently, neutrophil extracellular traps (NETs) were linked to the emergence of venous thrombi in the lungs.^138^ Further investigation revealed that neutrophil-associated CTCs, both in the 4T1 mammary tumor model and in patients with BC, displayed a pro-tumoral gene expression profile characterized by the enrichment of positive regulators of cell cycle progression and DNA replication. This pro-tumor phenotype contributes to enhanced metastatic capabilities. Consistent with these observations, using antibodies to block neutrophils reduced the incidence of bone metastasis. Conversely, upregulation of the granulocyte-colony-stimulating factor intensifies the clustering of TANs and CTCs, thereby exacerbating metastasis.^139^

Neutrophils play a dual role in shielding CTCs during immune surveillance in patients with BC. They inhibit the responsiveness of natural killer (NK) cells by attenuating signaling through cell surface receptors.^140^ Furthermore, neutrophils protect tumor cells from antitumor T-cell responses. An increase in T-cell-suppressive neutrophils has been systemically observed in mammary tumor models.^141^ Neutrophils derived from patients with melanoma and renal cell carcinoma exhibit elevated levels of ARG1, an enzyme that inhibits T-cell-mediated cytotoxic responses. Neutrophil depletion restores cytotoxic T-cell proliferation.^142,143^ Moreover, recent findings suggest a collaborative mechanism wherein CTCs activate platelets and neutrophils through direct cell-cell interactions, creating a protective network within the vasculature.^144^ This mutual activation facilitates the clustering of disseminated tumor cells with neutrophils and platelets and likely shields CTCs from mechanical and immune-mediated destruction during the metastatic process (Fig. 3).

#### Cancer cell extravasation and seeding in the distal tissue

Besides surviving in circulation, CTCs must navigate several steps to establish colonies in distant organs. These include adhesion to and transendothelial migration through the endothelium, ECM degradation, parenchyma invasion. Initial interactions between CTCs and the endothelium are crucial in the metastatic niche. Endothelial selectins, such as E-selectin, facilitate CTC tethering and rolling through ligand-receptor interactions.^145^ Adhesion is further stabilized by specific integrin pairings, notably αvβ3 integrin, on CTCs and their endothelial ligands.^146–148^

Adhesion is a critical preliminary step when CTCs invade tissues. Given that different tissues possess distinct homeostatic environments, CTCs utilize various strategies, each adapted to the specific conditions of the tissue in question, to ensure successful extravasation and seeding. This process entails distinctive molecular interactions and adaptations to the distinctive microenvironments of target tissues, thereby ensuring effective colonization and growth. Because the bone represents a primary organ frequently targeted by a multitude of cancers, this discussion will focus on elaborating on the phenomenon of bone metastasis in various tissues.

Chemokine signaling via the CXCL12-CXCR4 axis directs CTCs to CXCL12-rich bone sites.^149,150^ After attaching to the endothelium, CTCs must penetrate the vascular barrier to reach the bone marrow, secrete VEGF to increase vascular permeability, and aid in their transendothelial journey.^151^ Interactions with vascular lining cells, including pericytes, can either facilitate or impede CTC extravasation^152^ (Fig. 3).

In the bone microenvironment, stromal cell networks significantly affect CTC metastatic potential. Osteoblasts emit chemoattractants, such as receptor activators of nuclear factor-κB ligand (RANKL), attracting CTCs that express the RANK receptor to the bone niche.^153^ CTCs manipulate bone remodeling by inducing osteoclasts via parathyroid hormone-related protein (PTHrP) secretion and releasing cytokines and growth factors from the bone matrix to create a nurturing environment for CTC proliferation.^154^ Some CTCs may enter dormancy within the bone and be reactivated under favorable conditions.^155–158^

Enhanced oxidative phosphorylation in macrophages is linked to the secretion of pro-tumor factors, aiding tumor cell survival and EMT via pathways such as Wnt/β-catenin signaling at metastatic sites.^159–161^ Neutrophils colocalize with tumor cells at metastatic sites, facilitating their adhesion and arrest, especially in the lung and liver, thus promoting the retention of tumor cells.^162,163^ Studies on aggressive mammary tumors and TNBC specimens have shown elevated neutrophil-derived NETs in the lungs.^164^ NETosis enhances tumor cell adhesion to neutrophil monolayers, a process mitigated by inhibiting NET formation. NETs encapsulate adherent tumor cells, trapping them at distant sites and correlating with increased metastatic burden, whereas NET inhibition reduces metastasis in vivo.^164–166^ Moreover, transendothelial migration of CTCs is mediated by neutrophil MMP8/9, with inhibition or genetic ablation of these enzymes, thus reducing the metastatic burden in murine models^140^ (Fig. 3).

In tumor immunology, monocytes are categorized as pro-tumoral, classical, anti-tumoral, and non-classical. Classical monocytes enhance cancer cell invasiveness, as evidenced by their co-culture with human BC cells, resulting in elevated MMP9, TNF, and growth factor production.^167^ Dormant tumor cells actively recruit circulating monocytes, which facilitates extravasation. Ly6C+ monocyte recruitment is driven by CCL2 secretion, promoting BC cell extravasation into the lung tissue via VEGFA and MMP9.^168^ Similarly, Gr-1 + CD11b+ myeloid lineages contribute by releasing MMP9, disrupting endothelial monolayers, and enhancing vascular permeability.^169^ Collectively, these studies highlight the role of neutrophils and classical monocytes in regulating endothelial permeability, facilitating cancer cell extravasation, and bone colonization (Fig. 3).

#### Metastatic cancer dormancy, reactivation, and outgrowth

Metastatic cancer dormancy and reactivation are complex processes within a metastatic microenvironment. Disseminated tumor cells dynamically interact with local stromal cells, pivotal for transitioning tumor cells from a dormant state to active metastatic proliferation.

The maintenance of dormancy in metastatic cells depends on several mechanisms. Interactions with the ECM, including fibronectin,^170^ tenascin C, and periostin,^171^ are crucial for survival, with adhesion molecules, such as integrins, playing a pivotal role. Maintenance of the dormant state is facilitated by stress signaling pathways, including p38 MAPK and the unfolded protein response.^172^ Hypoxic conditions and their associated signaling pathways also contribute to the maintenance of cellular quiescence. Furthermore, dormant cells can evade immune detection, enabling them to survive in a non-proliferative state. These integrated mechanisms facilitate the persistence of dormant cells in a stable state until the conditions are conducive to reactivation and growth^173–176^ (Fig. 3).

In breast and prostate cancer models, prolonged systemic inflammation induces neutrophil infiltration and NET formation at metastatic sites. NETs, by remodeling the ECM component laminin, activate a cascade involving WNT signaling, integrin signaling, and the FAK/ERK/MLCK/YAP pathways, which awaken dormant tumor cells, thereby promoting their proliferation.^177–181^ In addition, chronic inflammation increases reactive oxygen species and promotes angiogenesis, which breaks the dormancy.^178–180^ Neutrophil accumulation in the lungs precedes significant tumor cell invasion, and systemic perturbation in myelopoiesis is evident in both mouse models and human BC.^96^ In melanoma, factors that inhibit macrophage migration stimulate Kupffer cells to secrete TGF-β, attract bone marrow-derived macrophages, and elevate fibronectin levels in the liver, underscoring the vital role of resident myeloid cells in metastasis.^182^ In mammary tumors, the myeloid lineage of the primary tumor influences distant pre-metastatic niches, where tumor-derived CCL2 fosters TAM accumulation and elevated IL1β secretion, thereby facilitating immunosuppression at metastatic sites.^183,184^

Metastasis-associated macrophages (MAMs) have recently been identified as distinct macrophage subpopulations and critical players in metastatic processes that facilitate the proliferation of metastatic cells. The reduction in metastatic outgrowth following macrophage depletion highlights the critical role of macrophages in metastasis.^185^ MAMs promote metastatic cell survival by activating the Akt pathway, which provides resistance to pro-apoptotic cytokines. Furthermore, MAMs interact with CTCs via integrins such as vascular cell adhesion molecule-1 (VCAM-1) to form protective clusters that improve cancer cell survival during migration.^186^ Gr-1 + CD11b+ monocytes promote the establishment of metastatic tumor cells in the lungs, particularly in breast tumor-bearing mice, through mechanisms such as PDGF-BB-induced angiogenesis and CCL9 production,^187^ which support tumor cell survival^188^ (Fig. 3).

## Mechanisms of organ tropism

The distinct genomic and epigenomic variation patterns observed across diverse tumor types and their subtypes, the specific molecules expressed by tumor cells, and the intricate interactions between these cells and the metastatic organ microenvironment collectively constitute a fundamental framework for understanding organ tropism mechanisms^189,190^ (Table 2). This intricate and sophisticated network offers several potential targets for developing targeted therapeutic strategies.^191^

### Bone metastasis

Bone is the preferred site for metastasis in several types of cancer, which is closely related to the unique microenvironment within bones, including high vascularization, hypoxic conditions, and a high local calcium concentration.^24,192,193^ The propensity for bone metastasis to predominantly affect the axial bones, such as the spine, pelvis, and ribs, rather than the distal bones, such as those found in the extremities, is significantly associated with the distribution of red bone marrow.^194,195^ The distinctive sinusoidal configuration of the skeletal vasculature endows bones with enhanced accessibility to CTC, thereby establishing them as primary targets for metastatic colonization.^11^ Furthermore, the bone exhibits a markedly hypoxic microenvironment, with prevailing oxygen tension frequently declining below 2%.^196^ This hypoxic microenvironment induces the activation of hypoxia-inducible factor (HIF) signaling in tumors, thereby triggering a cascade of events, including EMT, cell invasion, and angiogenesis. These processes facilitate the infiltration, metastasis, and colonization of tumor cells within the bone.^197^ Analysis of primary breast cancer specimens from bone metastasis has consistently demonstrated an elevation in the expression of HIFs, highlighting the critical role of hypoxia in driving organ tropism.^198^ In bone tissue, calcium levels typically range from 2 to 4 mmol/L, whereas in zones of active remodeling, they can reach concentrations of 8–40 mmol/L.^199^ Elevated local calcium concentrations can activate calcium-sensing receptors (CaSRs) in cancer cells, potentially amplifying proliferation, enhancing migratory capabilities, and blunting apoptotic signals.^200^ A distinctive attribute of CaSR in malignant cells is its inclination toward Gαs proteins, a deviation that results in the production of cAMP and PTHrP, further promoting tumor progression and dissemination.^201–203^

The concept of the “PMN” is important for understanding how specific secondary sites become the preferred locations for cancer metastasis. In the context of TNBC, the bone microenvironment is notably enriched with CXCL12 (also known as SDF-1) and insulin-like growth factor 1 (IGF-1), which are secreted by CAFs. These cytokines selectively drive the bone-tropic metastasis of cancer cells exhibiting elevated Src activity via stimulation of the PI3K-Akt pathway, which is pivotal in regulating cellular survival and motility.^204^ SCUBE2, a tumor-secreted glycoprotein, is a crucial facilitator of bone metastasis in luminal breast cancer, particularly during the initial stages of niche formation.^205^ SCUBE2 indirectly inhibits leukocyte-associated Ig-like receptor 1 (LAIR1) signaling, impairing NK cell function and promoting tumor cell persistence and growth within the bone. Exosomes are nanoscale vesicles secreted by tumor cells that serve as vital communicators between neoplastic cells and pre-metastatic niches, demonstrating a predilection to home organs expressing cognate ligands.^55^ Upon engagement, exosomal microRNAs (miRNAs) can modulate gene expression within target cells, thereby engineering a hospitable microenvironment conducive to the anchorage and proliferation of tumor cells.^206,207^ Furthermore, growth differentiation factor 15 (GDF15), secreted by prostate cancer cells, has also been identified as a factor contributing to the increased propensity for bone metastasis, as demonstrated in preclinical xenograft models.^208^

The proclivity for bone metastasis is inextricably linked to a vicious cycle that involves tumor cells and osteoclasts.^209^ Tumor cells secrete osteolytic substances, including PTHrP, IL-11, and Jagged 1, which induce bone resorption. These secretions activate the RANK/RANKL and Notch pathways, stimulating osteoclastogenesis and activation, exacerbating bone destruction, and providing a conducive environment for metastatic growth.^154,210,211^ Osteolysis in metastatic bone releases of key biological factors, including transforming growth factor beta (TGF-β), IGF-1, and calcium.^200,212,213^ These substances profoundly influence cancer cell growth, proliferation, and propensity for bone metastasis, thereby creating an environment conducive to the establishment and progression of skeletal lesions.

### Brain metastasis

Brain metastasis, a regrettable common occurrence among patients diagnosed with lung, breast, and melanoma cancers, is associated with unfavorable prognoses and reduced survival rates.^214^ Transmigration of tumor cells across the blood-brain barrier (BBB) through diverse mechanisms represents a critical step in the inception of brain metastasis. It is a determinant factor in the organotropism observed in cancer dissemination.^215^ A compromised BBB integrity, frequently associated with the upregulation of specific genes, plays a pivotal role in this process.^216,217^ For instance, the proteolytic action of cathepsin S, mediated by its interaction with the adhesion molecule JAM-B, can induce BBB leakage. Inhibition of cathepsin S expression markedly reduces the likelihood of brain metastasis.^218^ Brain metastases from triple negative or basal-type breast cancers frequently disrupt the BBB, in contrast to those from HER2/neu-positive breast cancer, which are inclined to maintain the BBB’s integrity. This phenomenon is closely associated with the differential expression of glucose transporter 1 (GLUT1) and breast cancer resistance protein (BCRP).^219^ Furthermore, elevated expression of adhesion molecules, including MUC1, VCAM1, and VLA-4, in breast cancer cells has been identified as a contributing factor in the facilitation of brain metastasis, thereby enhancing tumor cell adherence.^220^ Notably, primary tumor cells can also facilitate brain metastasis by exchanging exosomes, which are envelopes carrying miRNAs that communicate with pre-metastatic niches.^221^ Once cancer cells successfully colonize the brain, the BBB may transform its role, shifting from a protective barrier to an impediment against therapeutic interventions, thereby complicating treatment efficacy.^21^

The metabolic mechanisms of tumor cells are important for cancer invasion and metastasis. Different tumor types can result in significant variations in the metabolic characteristics of brain metastasis.^222,223^ Some tumors demonstrate a proclivity for anaerobic glycolysis, whereas others rely on oxidative phosphorylation (OXPHOS) for energy production.^224^ Given the distinctive energy storage and consumption mechanisms of the brain, tumor cells are compelled to adapt to their metabolic microenvironment.^225^ Genomic analysis of brain metastasis in melanoma has revealed that tumor cells can express genes related to the OXPHOS pathway at high levels.^226^ Additionally, inhibition of OXPHOS activity has been demonstrated to prevent melanoma brain metastasis in a mouse model.^226^ Furthermore, elevated expression of fatty acid-binding protein 7 (FABP7) in breast cancer is closely associated with a high incidence of brain metastasis. FABP7 facilitates a glycolytic phenotype and storage of lipid droplets, thereby enabling HER2-positive breast cancer cells to adapt more effectively to the relatively hypoxic and nutrient-restricted microenvironment of the brain.^227^

Neurons and glial cells work together to construct the PMN of the brain, and their interactions with cancer cells are crucial for promoting brain metastasis.^228^ Gamma-aminobutyric acid (GABA) is an inhibitory neurotransmitter that plays a critical role in the central nervous system.^229^ In the clinical analysis of HER2+ and TNBC brain metastasis, cancer cells have been observed to overexpress GABA-related proteins (such as GABAA receptors, GABA transporters, GABA transaminases, and glutamate decarboxylase). This overexpression influences organotropism of tumor metastasis.^230^ Glutamatergic neurons can form pseudo-tripartite synapses with breast cancer cells, a distinctive intercellular structure that directs glutamate signaling from neurons to cancer cells.^231^ This cross-cell interaction supports the survival and proliferation of breast cancer cells in the brain, thereby explaining the specific preference of these cells for this organ. Furthermore, brain metastatic tumor cells can deliver the second messenger cGAMP (cyclic GMP-AMP) to astrocytes via gap junctions, thereby activating the STAT1 (signal transducer and activator of transcription 1) and nuclear factor kappa B (NF-κB) signaling pathways within tumor cells through a series of cascades.^232^ These two signaling pathways are intimately associated with the growth, proliferation, and survival of tumor cells.^225,233^

### Liver metastasis

The liver is a highly vascularized organ that serves as a distal metastatic target for a multitude of solid tumors, including those of breast, pancreas, and colorectal origin.^234,235^ Tumor cells are disseminated to potential niches via the bloodstream, and the liver receives blood from both the hepatic artery (~25%) and portal vein (~75%), providing a direct pathway for tumor cells to reach the liver.^45,236^ Conversely, the low flow rate in the hepatic sinusoids allows for the retention and deposition of tumor cells in the liver, prolonging their retention time. Conversely, the high permeability of hepatic sinusoidal endothelial cells and the incomplete basement membrane facilitate tumor cell penetration of the vascular wall and subsequent entry into the liver parenchyma.^225,237^ CRC cells are more likely to be “captured” in the liver during the metastatic process than to remain in the peripheral blood. This increased propensity for liver metastasis is characteristic of CRC.^235,238^ Furthermore, the liver exhibits distinctive immune tolerance, particularly toward NK cells, which can impede the immune system’s capacity to eradicate tumor cells.^239,240^

Exosomes play a pivotal role in intercellular communication and are instrumental in PMN formation in the liver.^235^ In pancreatic cancer liver metastasis, macrophage migration inhibitory factor (MIF)-containing exosomes can specifically activate Kupffer cells in the liver under the mediation of αvβ5 integrin, thereby inducing the secretion of TGF-β and promoting tumor-specific liver metastasis.^182^ In addition, hepatocytes can respond to cytokines secreted by tumors, such as IL-6, by producing myeloid cell chemoattractants, such as serum amyloid A (SAA). They participate in formating an inflammatory and fibrotic microenvironment that favors the growth and dissemination of metastatic tumor cells in the liver.^234^ In addition, exosomes can carry CD39 and CD73, which inhibit T-cell function and aid tumor escape from immune surveillance.^241^ Neutrophils can release NETs, which contain DNA components that promote the proliferation and migration of tumor cells by activating CCDC25 (a cell cycle regulatory protein), thus facilitating liver metastasis.^242^

Tumor cells adapt to changes in the metastatic microenvironment of the liver through specific gene mutations or aberrant expression. *DAMTS10, NELL1*, and *RXFP3* are regarded as liver metastasis-specific genes, exhibiting mutations that are exclusive to liver metastatic regions but are absent in CRC lacking liver metastasis.^243^ Liver metastatic CRC cells upregulate the GATA6 transcription factor, increaseing the expression of aldolase B (ALDOB). This confers the ability to metabolize fructose and enhances the proliferative potential of tumor cells following metastasis.^244^ In the context of liver metastasis, elevated SERPINE2 expression has been observed to enhance epidermal growth factor receptor (EGFR) signaling, which is conducive to the proliferation of tumor cells.^245^ The expression of SERPINE2 is influenced by the status of DNA methylation, thereby indicating that epigenetic alterations may also affect the process of liver metastasis.^246^

### Lung metastasis

The lung has the highest incidence of metastasis, particularly in patients with breast cancer, melanoma, and thyroid cancer.^225,247^ The extensive vascular network of the lungs provides a conducive environment for tumor cells to adhere, extravasate, and establish micrometastasis. For example, thyroid cancer cells usually metastasize via the bloodstream, and because the lung is the terminal point of the superior vena cava system, this facilitates their metastasis to the lung.^45,248^ The typically high oxygen levels in the lung starkly contrast with the hypoxic environments of the bone and liver. Tumor cells that metastasize to the lungs must adapt their metabolic pathways to accommodate the microenvironment and mitigate oxidative damage.^249^ Examination of single-cell transcriptomes of breast cancer micrometastasis in the lung demonstrated elevated levels of OXPHOS activity, which contrasts with the energy production methods observed in primary breast cancer cells.^250^ Activation of the Notch signaling pathway promotes the expression of EMT-related genes, enabling breast cancer cells to acquire migratory and invasive capabilities that facilitate metastasis.^251^ For example, when breast cancer cells invade the lung, they produce tenascin C, which enhances Notch signaling.^252^ Secreted protein acidic and rich in cysteine (SPARC) derived from melanoma promotes lung metastasis by increasing vascular permeability and promoting the adhesion of tumor cells to vascular endothelial cells, mainly through VCAM-1-dependent mechanisms.^253^

Both breast and melanoma cancer cells express high levels of CXCR4 and CCR7 receptors, which enables them to migrate to the lungs in response to CXCL12 and CCL21 ligands.^254^ Exosomes released from CD103+ CSCs are enriched for miR-15b-3p, promoting the colonization and growth of clear cell renal cell carcinoma (CCRCC) cells in the lung and accelerating metastasis.^255^ EVs released by hepatocellular carcinoma (HCC) cells contain nidogen 1, which enhances angiogenesis and activates fibroblasts in the lungs, thereby contributing to the survival and proliferation of tumor cells.^256^

Osteosarcoma, a highly representative primary malignancy within bone and soft tissue sarcomas, primarily disseminates through the vascular system without involving the lymphatic system, with the lung often serving as its primary metastatic destination.^257^ The microenvironment plays a pivotal role in regulating the behavior of osteosarcoma cells, influencing not only their proliferation, quiescence, invasion, migration, and drug resistance characteristics but also contributing to the development of intrinsic tumor heterogeneity.^257^ By releasing EVs, osteosarcoma cells can remotely manipulate the lung environment, thereby predisposing a pre-metastatic niche for migrating tumor cells.^258^

Moreover, mesenchymal stem cells (MSCs) exhibit a profound connection with both the metastatic progression and therapeutic resistance of osteosarcoma.^259^ Specifically, EVs secreted by osteosarcoma cells, containing TGF-β, are capable of inducing MSCs to release IL-6, which in turn activates STAT3-mediated tumor progression pathways. This activation drives the formation of metastatic foci in the lungs and fosters the maturation of these MSCs.^260^ Furthermore, EVs originating from both osteosarcoma cells and metastatic niches possess the remarkable ability to reprogram myofibroblasts and osteosarcoma stem cells into fibrotic phenotypes, a process that is pivotal for metastatic colonization.^261,262^ In the osteosarcoma metastatic cascade, the cytoskeletal linker protein Ezrin provides essential scaffolding, thereby enhancing the survival of disseminated tumor cells under initial stress conditions in the lungs.^263^ Furthermore, the RANK-RANKL-OPG system has been identified as significant factors influencing the formation of the pre-metastatic niche in the lungs.^264^

### Other metastasis

A more profound understanding of tumor cell metastasis in the lymphatic system and peritoneum could facilitate the development of more efficacious treatment strategies and enhance our understanding of cancer staging.^191^ The structure of lymphatic vessels permits the passage of larger cells, and the slow flow rate of lymph provides opportunities for cancer cells to adhere to the walls of the lymphatic vessels.^265^ Lymph nodes serve not only as the initial destination for tumor cells following their departure from the primary tumor^266^ but also as pre-tumor niches, providing a protected and supportive environment for the proliferation of disseminated tumor cells.^267^ Furthermore, lymph nodes are typically located in key areas of the body, such as the neck, armpit, and groin, near many vital organs. This configuration facilitates the dissemination of cancer cells through the lymphatic system. Lymph nodes serve as sentinels of the immune system and exhibit rich nutrient and growth factor contents.^268^ However, cancer cells use immune evasion mechanisms, such as the upregulation of MHC-I and PD-L1 expression, to evade immune surveillance and proliferate within lymph nodes.^269^

Peritoneal metastasis is a common occurrence in ovarian cancer, whereby cancer cells that have detached from the primary tumor surface are carried by the peritoneal fluid and adhere in large numbers to the peritoneal surface.^270^ The presence of a substantial number of adipocytes in the peritoneum has been associated with the promotion of tumor cell growth through the release of lipids, cytokines (such as IL-8), and upregulation of fatty acid-binding protein 4 expression.^271^

## Targeting cancer metastasis

**Fig. 4:**
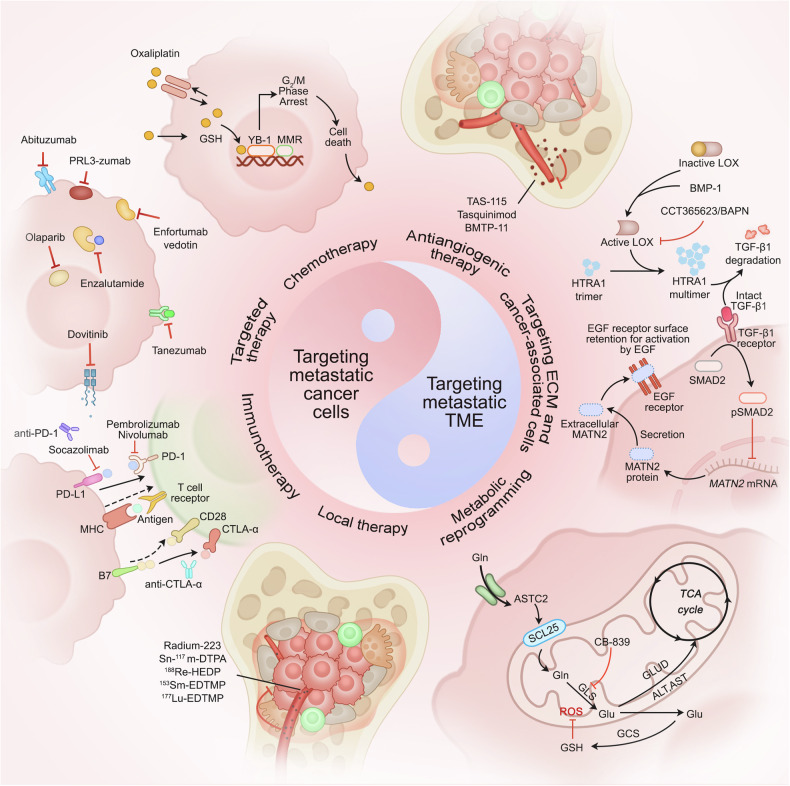
Therapeutic strategies targeting cancer metastasis. These strategies range from directly and precisely attacking metastatic cancer cells to delicately modulating the intricate TME, and further encompass personalized treatment plans for specific organ metastases. Specifically, these strategies integrate chemotherapy, targeted therapy, immunotherapy, local therapy, and combined therapy to achieve precise elimination of cancer cells. Additionally, anti-angiogenic therapy, targeting the ECM and tumor-associated cells, and regulating tumor metabolic mechanisms indirectly impact the TME, ultimately providing a more precise therapeutic approach for cancer metastasis

In oncology, the prevention or reversal of tumor metastasis is one of the most challenging and urgent clinical objectives. To address this challenge, scientists and clinicians have investigated the molecular mechanisms of tumor metastasis from a multitude of perspectives by directly targeting metastatic cancer cells. Crucially, cancer cells do not function in isolation but engage in a complex “symbiotic relationship” with their surrounding microenvironment. This underscores the need for targeting this environment as a pivotal therapeutic strategy. Treatment strategies for tumor metastasis are progressing toward greater precision, efficiency, and multi-target capabilities, offering promising avenues for advancing cancer care (Fig. 4) (Table 3).

**Table 3:** Therapeutic strategies targeting cancer metastasis

Therapeutic strategies	Therapeutic method	Mechanisms	Advantages	Disadvantages
Targeting metastatic cancer cells	Chemotherapy	Inhibit tumor growth or destroy tumors by chemical agents	Remarkable therapeutic effect for special cancer metastasis, such as lung metastasis	Adverse effects: nausea, vomiting, hair loss, bone marrow suppression, etc
	Targeted therapy	Interferes with specific molecular pathways associated with cancer cell growth, division, metastasis, and viability	High accuracy and improved therapeutic effectiveness	Adverse effects and limited population who is suitable
	Immunotherapy	Enhance the capacity of immune system to accurately identify and eradicate cancer cells	Low toxicity and strong lethality	Allergic reactions and infections.
	Local therapy	Surgical resection and external radiation therapy	Limited adverse effects and strong ‌ pertinence	Limited application scope‌, ‌ local injury and long treatment time
	Combined therapy	Combination of multiple methods	Incorporate the advantages of various treatment modalities	Adverse effects and high expense
Targeting metastatic TME	Anti-angiogenic therapy	Deprive tumor cells of essential nutrients and oxygen	Strong specificity	Affect wound recovery, hypertension and proteinuria
	Targeting the ECM	Remodeling the ECM to regulate the invasion and migration of cancer cells	Limited adverse effects and improved drug delivery efficiency	Drug resistance and high expense
	Targeting cancer-associated cells	Modulate the activity, function, and interactions of cancer-associated cells with cancer cells	High accuracy and improved therapeutic effectiveness	Adverse effects and limited population who is suitable
	Targeting metabolic reprogramming	Modulate glycolysis, glutaminolysis, one-carbon metabolism, and lipid metabolism	Limited adverse effects and improved therapeutic effectiveness	Drug resistance and high expense

### Targeting metastatic cancer cells

#### Chemotherapy

Chemotherapy is the conventional approach to cancer treatment. This entails the utilization of drugs that disrupt the growth and division of cancer cells, ultimately leading to their eradication or proliferation inhibition. Among the many metastatic cancers, metastatic lung cancer is one of the solid tumors most responsive to chemotherapy.^272^ Platinum-based drugs combined with gemcitabine, paclitaxel, docetaxel, or vinorelbine represent primary therapeutic options for patients with metastatic lung cancer. Other agents, including docetaxel, erlotinib, and pemetrexed, have received clinical approval as second-line treatments.^273^ Oxaliplatin exhibits a broad spectrum of anticancer activity and was approved for the treatment of colorectal cancer. It enters cells through passive diffusion and active transport mechanisms. It undergoes non-enzymatic biotransformation with nucleophilic reagents such as glutathione (GSH), converting it into more reactive species. These reactive forms of oxaliplatin subsequently form covalent DNA adducts with DNA, which inhibit DNA synthesis and transcription, ultimately leading to cellular apoptosis.^274^

In cancer brain metastasis, the BBB presents a significant challenge for the diffusion of chemotherapeutic drugs into the brain. In addition to direct injection into the cerebral circulation, the use of drugs capable of traversing the BBB, such as the alkylating agent temozolomide, has emerged as the preferred strategy.^275,276^ Notably, the expression level of P-glycoprotein in blood vessels at metastatic brain sites is lower than that in normal vessels and primary brain tumors.^277^ This influences drug efflux and results in increased pharmacological concentrations of paclitaxel in metastatic brain tumors. This observation indicates that metastatic brain tumors may be more susceptible to chemotherapy than primary brain tumors.^278^

Despite the efficacy of chemotherapeutic agents against rapidly proliferating cancer cells, they confront numerous challenges in suppressing metastatic cancer cells, including the emergence of drug resistance. During the process of chemotherapy resistance, CSCs can persist in a dormant state for decades after initial treatment, evading elimination by chemotherapy and retaining their self-renewal and differentiation properties, which are critical factors underlying recurrence and therapeutic resistance.^279^ Recent studies have indicated that CSCs become enriched following chemotherapy or radiotherapy, suggesting that treatment may induce reprogramming or dedifferentiation of normal cancer cells into those with enhanced CSC characteristics.^280,281^ During chemotherapy, CSCs in various cancers express abundant ATP-binding cassette transporters, which efflux chemotherapeutic drugs, leading to drug resistance.^282–284^ Additionally, the tumor microenvironment fosters the growth and proliferation of CSCs, contributing to metastasis and drug resistance.^285^ In colorectal cancer cells, exosomes secreted by CAFs trigger CSC activation, resulting in resistance to 5-fluorouracil.^286^ Trastuzumab resistance has been demonstrated to be mediated by an IL-6 inflammatory loop in HER2+ breast cancer CSCs.^287^

#### Targeted therapy

Targeted therapy is of paramount significance in the management of metastatic cancer cells, given that these cells often escape the confines of the primary tumor and disseminate through the bloodstream or lymphatic system to distant sites within the body. Although traditional chemotherapeutics can inhibit cancer cell growth to a certain extent, they often lack specificity and inadvertently damage healthy cells. Targeted therapies are designed to interfere with specific molecular pathways associated with cancer cell growth, division, metastasis, and viability. Targeted therapies can enhance treatment efficacy while minimizing adverse effects by recognizing and acting on these unique molecular targets exclusive to cancer cells.

These agents typically target receptors on cancer cell surfaces, signaling pathways, or intracellular enzymes. In patients with breast cancer and brain metastasis, HER2-targeted therapies, including trastuzumab, pertuzumab, neratinib, tucatinib, and pyrotinib, have demonstrated efficacy in the treatment of brain cancer.^288–293^ In patients with HER2-positive breast cancer who have undergone treatment with anthracyclines or taxanes and have developed brain metastasis, the combination of pyrotinib with capecitabine has been demonstrated to yield a superior median progression-free survival (PFS) of 11.1 months compared with 4.1 months in the placebo arm.^294^ Moreover, the combination of tucatinib with trastuzumab and capecitabine demonstrated enhanced therapeutic efficacy, with augmented rates of central nervous system responses and prolonged PFS.^295^ In patients with HER2-negative, hormone receptor-positive breast cancer and brain metastasis, the CDK4/6 inhibitor abemaciclib demonstrated an intracranial clinical benefit rate of 24%, indicating its potential for further investigation.^296^ Inhibition of EGFR represents a promising therapeutic strategy for metastatic lung cancer.^297^

EGFR-specific tyrosine kinase inhibitors, including erlotinib, cetuximab, and gefitinib, reversibly inhibit EGFR by blocking its intracellular ATP-binding domain, thereby effectively treating metastatic lung cancer with EGFR mutations.^298–300^ Furthermore, ALK inhibitors such as crizotinib, ceritinib, and alectinib have demonstrated efficacy in treating brain metastasis in non-small cell lung cancer (NSCLC).^301^ Dasatinib, a Src kinase inhibitor that impedes cancer cell growth, is another promising antagonist.^302^

Inhibition of the PI3K-Akt-mTOR pathway may be a promising therapeutic option for approximately 70% of breast cancer patients with bone metastasis.^303^ The ability of these agents to effectively cross the BBB remains a significant challenge. However, GDC-0084 and GDC-0068 have demonstrated the capacity to overcome this hurdle by inhibiting the PI3K-Akt-mTOR pathway and exhibiting potential to treat breast cancer brain metastasis.^303,304^

Additionally, dovitinib, an oral FGFR inhibitor, demonstrated moderate antitumor activity in patients with mCRPC, with controllable toxicity. Patients who do not undergo chemotherapy may benefit more from dovitinib than from docetaxel.^305^ Tanezumab, a NGF inhibitor, is commonly used to treat bone and joint arthritis and to alleviate chronic lower back pain. In phase III clinical trial for severe bone metastasis cancer pain, tanezumab demonstrated a more significant improvement in pain site intensity than opioid drugs at 8 weeks.^306^ Enfortumab vedotin, an antibody-drug conjugate targeting nectin-4, has been demonstrated to be safe and effective in treating metastatic urothelial carcinoma, with an objective response rate of 44%.^307^

#### Immunotherapy

Immunotherapy, which is directed toward metastatic cancer cells, represents a revolutionary advancement in cancer treatment. This is achieved by leveraging and enhancing the capacity of a patient’s immune system to accurately identify and eradicate cancer cells that have disseminated to other regions of the body. This approach overcomes the limitations of conventional therapies and offers a promising avenue for patients with metastatic cancer who are unresponsive or have developed resistance to traditional chemotherapy, radiotherapy, and similar treatments.

Immunotherapy exerts its effects through a multitude of mechanisms, with immune checkpoint inhibitors garnering particular attention. These agents remove the immunosuppressive “brakes” imposed by cancer cells on the immune system, such as the PD-1/PD-L1 pathway, thereby unleashing the full potential of immune cells like T cells to recognize and attack cancer cells more efficiently.^308,309^ Notably, the PD-1/PD-L1 pathway serves as a pivotal immune regulatory axis, with drugs such as pembrolizumab and nivolumab targeting PD-1. These drugs effectively manage brain metastasis in patients with melanoma and NSCLC and alleviate central nervous system symptoms.^310,311^ Socazolimab (ZKAB001), a PD-L1-specific monoclonal antibody, has been demonstrated to be safe in nonprogressive localized high-grade osteosarcoma and beneficial for PD-L1-positive and microsatellite instability-high subgroups of patients.^312^ However, not all anti-PD-1 monoclonal antibodies (mAbs) exhibit therapeutic effects. Pembrolizumab has been shown to improve distant metastasis-free survival in patients with stage IIB and IIC melanoma after surgical resection.^313^ However, the same effect has not been observed in patients with advanced osteosarcoma.^314^ Following anti-angiogenic therapy for metastatic clear cell RCC, patients with bone metastasis treated with nivolumab exhibited a poorer prognosis with lower PFS and objective response rates.^315^ Moreover, ipilimumab, which targets the CTLA-4 checkpoint, when combined with nivolumab in the treatment of melanoma brain metastasis, significantly enhances both the response rates within the central nervous system and objective response rates within the intracranial region.^316^

Cellular therapies such as CAR-T cell therapy exhibit considerable promise. For example, Priceman et al. optimized the 4-1BB co-stimulatory domain in HER2-CAR-T cells. Compared to CD28 co-stimulation, this modification demonstrated a reduction in T-cell exhaustion phenotypes, enhanced proliferation, and potent antitumor activity in breast cancer brain metastasis models.^317^

#### Local therapy

In addition to the specific targeting of tumor cells, therapeutic interventions for metastatic tumors include surgical resection, and minimally invasive and noninvasive modalities.

In the context of oligometastatic disease, surgical procedures, such as lung resection,^318^ hepatectomy for liver metastasis,^319^ and craniotomy for brain metastasis,^273^ have demonstrated that current surgical techniques remain viable options for treating metastatic disease. However, given the complexity of metastatic behavior and the potential involvement of multiple organ systems, minimally invasive or noninvasive approaches have emerged as the primary choice among healthcare professionals.^320^

External beam radiation therapy (EBRT) uses radiation beams that traverse normal tissues and adjacent organs to target specific pathological sites. It encompasses a variety of techniques, including radiofrequency ablation (RFA), stereotactic body radiation therapy (SBRT), three-dimensional conformal radiation therapy (3D-CRT), and hypofractionated stereotactic ablative radiotherapy (HSRT).^321^ The advent of image-guided technology has led to a surge in the popularity of RFA for hepatic metastasis. This approach has demonstrated efficacy in controlling tumors with lesion sizes < 3 cm and ablation margins > 5 mm, with long-term local control rates > 90%.^322^ Similarly, in a study of over 1,000 patients with lung metastasis treated with RFA, the four-year local control rate was 89%, with superior outcomes observed in smaller tumors.^323^ Stereotactic ablative body radiation therapy (SABR) is an effective treatment for a wide range of lesions, including lung, liver, and bone metastasis, as evidenced by robust data from various clinical settings.^324,325^ HSRT improves local control through fractionated high-dose regimens, resulting in enhanced 5- and 10-year overall survival (OS) rates in breast cancer patients with oligometastasis. The number of lesions may influence the risk of recurrence, necessitating further research to identify patients with breast cancer who would potentially benefit from metastasis-directed radiotherapy.^326^ Notably, radiation therapy alters the blood cytokine profile, thereby mediating analgesia in bone metastasis through the modulation of cytokine production. Several factors, including MIP-1δ, MCP-2, TIMP-1, RANTES, IGFBP3, and TNF-α, have been observed to undergo significant changes both before and after radiotherapy. These changes may play a role in the mechanisms underlying pain related to cancer metastasis.^327^

Magnetic resonance-guided focused ultrasound (MRgFUS, also known as MRgHIFU) uses focused ultrasound beams to generate thermal, mechanical, and cavitation effects within soft tissues, thereby rapidly heating the target area to achieve tissue coagulation and necrosis.^328,329^ Extensive research has been conducted on MRgFUS to treat a range of conditions, including uterine fibroids, osteoid osteoma, essential tremors, and cancers of the breast, prostate, liver, pancreas, and bone metastasis.^330,331^ The therapeutic mechanism of MRgHIFU in cancerous bone metastasis is primarily periosteal nerve ablation.^332,333^ A prospective, open-label, non-randomized Phase II study comparing MRgHIFU and EBRT for bone metastasis revealed comparable overall response rates and quality of life scores at one month, accompanied by a diminished incidence of adverse events in the MRgHIFU cohort.^331^

#### Combined therapy

Comprehensive therapy incorporates the advantages of various treatment modalities, addressing the processes of cancer cell proliferation and invasion holistically and synergistically. This approach emphasizes not only the direct eradication of cancer cells but also the restoration and enhancement of the patient’s immune system function and modulation of the TME, with the ultimate goal of achieving comprehensive control over metastatic cancer.

Comprehensive therapy typically encompasses a combination of targeted therapy, immunotherapy, chemotherapy, and radiotherapy, among other modalities. A clinical trial (RTOG 0320) observed an increased risk of cytotoxicity when whole-brain radiation therapy (WBRT) was combined with EGFR tyrosine kinase inhibitors (TKIs), specifically erlotinib or temozolomide. Nevertheless, other studies have demonstrated the efficacy and safety of EGFR-TKIs with WBRT for the treatment of brain metastasis from advanced lung cancer.^334^ Compared with erlotinib monotherapy, patients who received stereotactic radiosurgery (SRS) or WBRT demonstrated similar OS rates but a longer time to intracranial progression.^273,335^ Androgen deprivation therapy (ADT) is frequently used to improve patient survival in the management of metastatic prostate cancer.^336^ In a comparative study, Kyriakopoulos et al. examined the outcomes of chemotherapy with hormonal therapy versus ADT in patients with extensive-disease prostate cancer. The researchers defined high-volume disease as the presence of visceral metastasis and/or ≥4 bone metastasis with at least one outside the spine and pelvis. The findings revealed that docetaxel prolonged OS in patients with high-volume disease, but not in those with low-volume disease.^336^ In a study of patients with metastatic castration-resistant prostate cancer (mCRPC), Heery et al. investigated the efficacy of a therapeutic vaccine, PSA-TRICOM, combined with the radiopharmaceutical, samarium-153-ethylenediamine tetramethylenephosphonate (Sm-153-EDTMP). The study demonstrated that combination therapy resulted in improved PFS, as well as a trend toward enhanced PSA decline and PSA-specific T-cell responses, compared to Sm-153-EDTMP alone.^337^

### Targeting metastatic tumor microenvironment

#### Antiangiogenic therapy

Antiangiogenic therapy represents a precise therapeutic strategy that targets the vital processes of angiogenesis during tumor growth and metastasis. Angiogenesis is a pivotal factor in tumor progression in the metastatic TME. Cancer cells secrete a multitude of angiogenic factors (such as VEGF and FGF), which prompt the proliferation and migration of adjacent vascular endothelial cells. These cells form novel vascular networks, thereby supplying crucial nutrients and oxygen to tumors.^338^ By inhibiting the activity of angiogenic factors within the TME, this therapy impedes the formation and maturation of new blood vessels, thereby depriving tumor cells of essential nutrients and oxygen, which ultimately inhibit their growth, invasion, and metastasis.^339^

Antiangiogenic therapy uses specific inhibitors, such as bevacizumab and anlotinib, to interrupt the actions of angiogenic factors or to directly act on vascular endothelial cells, disrupting their proliferation and migration and suppressing tumor angiogenesis. Early studies in metastatic CRC have demonstrated improved median OS (20.3 months with bevacizumab vs. 15.6 months without; *P* < 0.001) and PFS; 10.6 vs. 6.2 months; *P* < 0.001) for the bevacizumab arm compared to traditional triplet regimens including 5-fluorouracil (5-FU), irinotecan, and leucovorin calcium (5-FU/LV/Irinotecan). In contrast, randomized clinical trials and long-term follow-up have demonstrated that bevacizumab does not confer a survival benefit in patients with metastatic breast cancer and is associated with a significant increase in severe adverse effects. Consequently, the FDA withdrew its approval for this indication, although Medicare and Medicaid Services continue to support the use of bevacizumab as a first-line treatment for metastatic breast cancer.^340^

Aflibercept, an engineered VEGF receptor, has been approved for Phase III clinical trials of metastatic NSCLC and pancreatic CRC.^273^ TAS-115 specifically targets VEGFR2 and completely inhibits both MET and tumor progression by blocking angiogenesis.^341^ Ramucirumab, a human IgG1 antibody that binds to both HER2 and VEGFR, offers limited benefits when combined with docetaxel in the treatment of certain advanced digestive system malignancies.^342^ However, it also provides a promising avenue for antibody-based therapies.^343^ Additionally, Taquimmod specifically targets S100A9, influencing the tumor-infiltrating myeloid cells in such a way that they undergo a phenotypic transformation. This change converts them from pro-angiogenic and immunosuppressive M2-like TAMs into pro-inflammatory M1-like macrophages, which have immunomodulatory, anti-angiogenic and metastatic inhibitory effects.^344^

#### Targeting the ECM

The ECM, a critical element of the TME, provides physical support to tumor cells and regulates their biological behavior through an array of growth factors, proteolytic enzymes, cytokines, and other mediators.^345^ During the process of tumor metastasis, remodeling and degradation of the ECM facilitate the invasion and migration of tumor cells, thereby promoting disease progression.^345^ Consequently, therapeutic strategies that target the ECM have been designed to impede tumor metastasis by intervening in this complex process.

Extracellular heat shock proteins (HSPs) play a pivotal role in ECM remodeling and augmentation of MMP activity, which is crucial for tumor metastasis.^346^ AUY922, an inhibitor of HSP90, exemplifies this approach by reducing fibronectin secretion into the ECM and impeding prostate cancer invasion.^347^

Lysyl oxidase (LOX) is another crucial enzyme involved in ECM remodeling. LOX inhibitors have been the subject of extensive preclinical research.^348^ Among these, β-aminopropionitrile (BAPN) and the aminomethyl thiophene-based inhibitor CCT365623 effectively suppress the migration and invasion of breast cancer cells.^349,350^ Furthermore, PAT-1251/GB2064, a highly selective LOXL2 inhibitor, has shown promise in reducing collagen accumulation and inhibiting tumor growth in preclinical settings.^351^ CCT365623 disrupts HTRA1 multimerization, activates TGF-β1 signaling, suppresses MATN2 expression, inhibits EGFR surface retention, and attenuates EGFR signaling. Ultimately, akin to BAPN or LOX gene ablation, CCT365623 downregulates MATN2, impeding EGFR plasma membrane localization in tumors and inhibiting tumor growth and metastasis in mice.^352^

Integrins, which are highly expressed in solid tumors, serve as signaling hubs that transmit messages from the interior to the exterior of the cell. This process modulates cell-ECM interactions, which alter cell adhesion, migration, and ECM properties.^63^ Notably, integrin αvβ3 is more prevalent in metastatic tumors than in primary pancreatic and breast cancers.^353^ This enhances tumor migration and metastasis by recruiting Src kinase. Selective expression of integrin αvβ3 at metastatic sites offers a promising avenue for enhancing the delivery of chemotherapeutic agents to bone-resident breast cancer cells with greater precision.^354^

Given the pervasive role of the ECM across various tumor types, therapies targeting the ECM have broad application prospects. However, it is essential to recognize that the ECM is a highly complex and dynamic system, and its composition and structure vary significantly among tumor types, stages, and even individuals. Therefore, precision medicine requires careful consideration of these differences when developing ECM-targeted therapeutics.

#### Targeting cancer-associated cells

Targeting cancer-associated cells within the TME, which encompasses CAFs, represents a promising approach for impeding tumor metastasis and progression. This can be achieved by modulating the activity, function, and interactions of these cells with tumor cells.

CCL5, produced by CD4 and CD8 T lymphocytes in liver metastasis of colorectal cancer, elicits protumorigenic effects via CCR5 signaling. This fosters monocyte recruitment and M2 polarization, enhances CAF expansion, and potentiates the TGF-β-mediated killing of CD8 T cells by Tregs. Notably, the CCR5 antagonist maraviroc, which inhibits CCR5, has been linked to the repolarization of tumor-associated macrophages, emerging as a promising avenue for further scientific and clinical exploration.^355^

Given the established correlation between the crosstalk of metastatic cancer cells and their microenvironment in the progression of cancer metastasis, there is growing emphasis on developing therapeutic strategies that target this microenvironment or the crosstalk itself. For example, silibinin impedes the growth of brain metastasis by targeting STAT3 in tumor-associated astrocytes, thereby reducing their crosstalk with cancer cells and microglia.^356,357^

Therapeutic strategies targeting CAFs offer notable advantages, as they not only directly impact crucial cellular components of the TME but also indirectly inhibit tumor growth and metastasis by influencing the entire microenvironment. Furthermore, given the critical roles that CAFs play in a multitude of tumor types, this approach offers significant potential for broad-spectrum applications.

#### Targeting metabolic reprogramming

Metabolic reprogramming of tumor cells is characterized by aberrant uptake and utilization of nutrients, such as glucose and glutamine, as well as abnormal accumulation and excretion of metabolic byproducts. This phenomenon is the hallmark of malignant transformation.^358^ This reprogramming satisfies the energetic demands of rapid tumor cell proliferation and influences their ability to invade, migrate, and evade immune surveillance by altering metabolite composition within the microenvironment.^359^ Consequently, therapeutic strategies targeting metabolic reprogramming seek to impede tumor metastasis and progression by modulating glycolysis, glutaminolysis, one-carbon metabolism, and lipid metabolism.

The proliferation and metastasis of tumor cells are closely associated with aberrant glycolysis.^360^ The glycolytic inhibitor 2-deoxy-D-glucose (2-DG) has been demonstrated to reduce the invasiveness of 5-FU-resistant CRC cells by downregulating glycolytic enzyme expression, while impairing the secretion of EMT-related cytokines and inactivating integrins, MMP-10, and MMP-17.^361^

Tumor cells utilize glutamine as a fuel source for the tricarboxylic acid (TCA) cycle, and targeting intermediates of the TCA cycle involved in glutaminolysis has proven to be an effective anticancer approach.^362^ New specific glutaminase (GLS) inhibitors, including CB-839 selenadiazole-derivatives CPD-20 and CPD-23, have been observed to demonstrate enhanced uptake in tumor cells and exhibit higher anticancer activity against CRC cells. CPD-20 and CPD-23 exhibit improved cellular and tumor accumulation, enhanced GLS inhibition, elevated ROS induction, and a better effect on eliminating cancer cells.^363^ Additionally, when CB-839 was combined with erlotinib in a dual therapy for mouse NSCLC xenografts, rapid tumor regression was observed in vivo. This combination simultaneously hindered cancer cells’ utilization of Gln and Glc, disrupted redox homeostasis, and induced autophagy to combat cancer.^364^ Likewise, CB-839 demonstrated notable antitumor activity in two xenograft models of TNBC cell lines, both as a monotherapy and in conjunction with paclitaxel.^365^

Abnormalities in lipid metabolism have been linked to several tumorigenic processes, including cancer progression and metastasis.^366^ Luteolin, functioning as a fatty acid synthase inhibitor, obstructs the de novo biosynthesis of long-chain fatty acids and exerts its anticancer effects in CRC by modulating a multitude of tumor signaling pathways, including IGF-1, Keap1-Nrf2-ARE, and Wnt-β-catenin.^367^

One-carbon metabolism is a pathway that generates one-carbon units that are essential for nucleotide synthesis, methylation, and NADH/NADPH production. These processes sustain a high proliferation rate of tumor cells.^368^ Methotrexate and chemotherapeutic agents that target key enzymes involved in one-carbon metabolism, such as dihydrofolate reductase and thymidylate synthase, are clinically utilized to treat a range of cancers, including CRC.^358^

### Targeting organ-specific metastasis

#### Targeting bone metastasis

A multifaceted approach is essential for the effective management of bone metastasis, encompassing the use of monoclonal antibodies, TKIs, hormone-related medications, bisphosphonates, small molecules, and radiopharmaceuticals.

In the context of targeted therapy for bone metastasis, denosumab, a monoclonal antibody, has demonstrated particular efficacy by specifically binding to and inhibiting RANKL, thereby impeding osteoclast-mediated bone resorption. It is the standard treatment for cancer patients with bone metastasis.^369–372^ Furthermore, biosimilars of denosumab, such as HS-20090 and QL1206, provide novel therapeutic options for patients with bone metastasis.^373,374^ Abituzumab, a pan-αv integrin inhibitor, has demonstrated promising PFS and a low cumulative incidence of bone lesion progression in asymptomatic or patients with mildly symptomatic metastatic castration-resistant prostate cancer (mCRPC). These findings highlight the specific activity of abituzumab in prostate cancer-related bone diseases and warrant further investigation.^375^

Cabozantinib, an oral TKI, has been demonstrated to reduce osteoblast proliferation and enhance osteoblast activation. This is achieved by inhibiting the MET and VEGFR-2 rearrangements that occur during the transduction of osteoblasts.^376,377^ In addition, it affects tumor cells and the TME.^378^ Nevertheless, the clinical efficacy of other TKIs remains to be elucidated.^379,380^

ADT is a fundamental component of prostate cancer management, particularly in metastatic and advanced stages. Enzalutamide, an oral androgen receptor antagonist, effectively halts receptor nuclear translocation upon binding, thereby disrupting androgen signaling.^381^ It markedly enhances outcomes for bone disease in both low- and high-volume settings^382^ and displays a favorable efficacy and safety profile in patients aged 75 years and above.^383^ However, it should be noted that patients with prostate cancer undergoing ADT may experience early bone loss (within three months),^384^ thereby increasing the risk of osteoporotic fractures.^385^

Bisphosphonates represent another essential class of bone-modifying agents (BMAs). These agents are analogs of pyrophosphate that bind to hydroxyapatite binding sites on bone, thereby inhibiting osteoclast-mediated bone resorption. In addition, they regulate osteoclast function by reducing the development, recruitment, and promotion of osteoclast progenitor cells as well as promoting osteoclast apoptosis.^386,387^ Bisphosphonates have been approved for the prevention and treatment of SREs associated with solid tumor bone metastasis.^386^ Nevertheless, long-term use of zoledronic acid in patients with bone metastasis requires careful consideration.^388^

Advances in our understanding of the mechanisms underlying bone metastasis have led to the identification of several key molecules that may represent promising therapeutic targets. Elevated expression of phosphatase of regenerating liver-3 (PRL-3) in tumor tissues has been demonstrated to contribute to metastasis. A Phase 1 clinical trial demonstrated the safety and tolerability of PRL3-zumab in advanced tumors, thereby encouraging further advancement of PRL-3-targeted therapies.^389^ Inhibition of the poly(ADP-ribose) polymerase (PARP) isoforms (PARP-1, -2, -3) has been demonstrated to reduce tumor growth in vitro and in vivo in human cancer models. In Phase III trials, olaparib, a PARP inhibitor, demonstrated significantly prolonged radiographic PFS in patients with mCRPC compared to enzalutamide or abiraterone.^390^ Tumor vascular receptors represent attractive targets for ligand-directed drug discovery and development. BMTP-11, developed by Pasqualini et al., targets bone marrow tumors by binding to the ligand-binding motif of interleukin-11 receptor α (IL-11Rα) in the human tumor vasculature, thereby inducing apoptosis.^391^

Radioisotopes release α, β, γ, and other rays, which cause ionization, generation of free radicals, DNA strand breaks, and ultimately, cancer cell apoptosis. Clinical trials on bone metastasis have utilized radium-223, Sn-117, rhenium-188, lutetium-177, and samarium-153 to demonstrate antitumor effects and pain relief. Radium-223 is primarily used in clinical trials to treat bone metastasis and exhibits antitumor and analgesic effects.^392,393^ In clinical practice, radium-223 reduces the length of hospital stay, improves survival without symptomatic skeletal events (SSEs), and enhances health-related quality of life (QOL) in patients with mCRPC.^394^ Analyses of the EuroQoL 5D and Functional Assessment of Cancer Therapy-Prostate instruments revealed that the survival benefits associated with radium-223 administration resulted in notable improvements in QOL. These benefits encompass an increased proportion of patients with castration-resistant prostate cancer experiencing enhanced QOL and a slower rate of decline in QOL.^395^ Sn-117 meta-diethylenetriaminepentaacetic acid (DTPA) (Sn-^117^m-DTPA) is a radioactive isotope of tin developed as a radioactive drug.^396^ Compared to radium-223, Sn-^117^m-DTPA appears to have lower bone marrow toxicity. A phase II clinical trial is underway to validate the efficacy of Sn-^117^m-DTPA for pain relief and antitumor activity in patients with mCRPC.^397^ Rhenium-188 was obtained from the ^188^W/^188^Re generator and involved fewer radiation protection issues than ^131^I did. ^188^Re-HEDP is safe and well tolerated, is effective in relieving pain in patients with painful bone metastasis from lung cancer, and improves the overall quality of life.^398^ Samarium-153 is a highly bone-seeking radioactive drug, similar to bisphosphonates, linked to ethylenediamine tetramethylene phosphonic acid (EDTMP).^399^ In advanced stages, requiring palliative treatment for bone metastasis, intravenous injection of ^153^Sm-EDTMP represents a potential option for managing cancer pain in patients who are intolerant or resistant to drug therapy. Patients receiving samarium therapy require fewer or no analgesics and are more cost-effective.^400^ In bone pain caused by multifocal myeloma or mixed lytic metastatic lesions, ^153^Sm-EDTMP and ^177^Lu-EDTMP demonstrated high affinity for lesions. As measured using the Edmonton Symptom Assessment System, ECOG performance status, and a numeric rating scale, ^153^Sm-EDTMP and ^177^Lu-EDTMP have been demonstrated to be safe and effective radioactive drugs for the relief of cancer pain caused by metastatic lesions with myeloma or mixed lytic characteristics.^401^

EBRT enhances prognosis and reduces SREs in patients with asymptomatic bone metastasis. The Phase II trial conducted by Gillespie et al. demonstrated the efficacy of EBRT in reducing the risk of SREs, and hospitalizations, and improving OS in patients with high-risk, asymptomatic bone metastasis.^321^ EBRT is recognized as the standard treatment for alleviating pain associated with symptomatic bone metastasis, offering an effective and commonly adopted approach.^321^ When formulating treatment plans, it is essential to consider the unique circumstances and characteristics of each patient. Further research and trials must validate the therapeutic outcomes and provide more guidance for clinical practice.

#### Targeting brain metastasis

Whole-brain radiotherapy (WBRT), surgical intervention, and stereotactic radiosurgery are the preferred options for the localized treatment of brain metastasis.^402^ A review of past studies has shown that surgical intervention can effectively delay recurrence in certain patients with brain metastasis and thereby improve their median survival rates.^403,404^ The combination of surgery and WBRT has been demonstrated to significantly prolong the time to recurrence and markedly improve median survival, thereby building upon the benefits of WBRT alone.^405,406^

In addition to localized therapies, systemic treatments such as chemotherapy and targeted therapy are also important in the management of brain metastasis. However, traditional cytotoxic chemotherapy has exhibited limited efficacy in the treatment of brain metastasis owing to the BBB.^407^ Targeted therapies designed to target specific mutations in lung cancer, breast cancer, and melanoma have emerged as important tools in the treatment of brain metastasis.

TKIs, such as erlotinib, gefitinib, and osimertinib, which target EGFR mutations in NSCLC brain metastasis, have demonstrated response rates exceeding 50%, effectively extending median survival.^408–410^ In cases of brain metastasis associated with ALK rearrangement in NSCLC, alectinib, ceritinib, and brigatinib, which demonstrate effective penetration of the BBB, show promise and warrant further investigation.^411–413^

Among patients with breast cancer and brain metastasis, those with HER2-positive status are more likely to have a higher incidence of brain involvement. Targeted therapies tailored to the molecular subtypes of primary breast cancers have been developed. In HER2-positive brain metastasis, a range of anti-HER2 agents has been the subject of clinical investigation, including monoclonal antibodies (e.g., trastuzumab and pertuzumab), antibody-drug conjugates (e.g., T-DM1), and small-molecule TKIs (e.g., lapatinib, neratinib, tucatinib, and pyrotinib).^414^ Preclinical trials demonstrated the limited activity of lapatinib as a single agent but exhibited good performance in combination with capecitabine, achieving over 65% response rates in radiotherapy-naive patients and 20% in radioresistant patients.^415–417^ In a phase 3 study involving 991 patients who had undergone treatment for HER2-positive brain metastasis, trastuzumab demonstrated superior efficacy and safety outcomes compared to capecitabine plus lapatinib.^288^ The combination of HER2-targeted inhibitors with cytotoxic chemotherapy demonstrated some efficacy in clinical trials; however, concerns have been raised regarding the response instability and frequency of adverse events. Thus, further efforts must be made to optimize efficacy and reduce toxicity.^418^

In patients with melanoma and brain metastasis, mutations in BRAF and NRAS indicate potential for targeted therapeutic approaches.^419^ Early clinical studies have demonstrated the efficacy and tolerability of dabrafenib and trametinib in patients with BRAF-mutated brain metastasis, with approximately one-third of the patients with unresectable or metastatic melanoma experiencing long-term benefits.^420,421^ Moreover, MEK inhibitors have been combined with BRAF inhibitors, including encorafenib plus binimetinib and vemurafenib plus cobimetinib, and have demonstrated effective clinical responses.^422,423^

#### Targeting liver metastasis

Despite advancements in chemotherapy that have led to improved survival rates, only 10–20% of patients with liver metastasis are eligible for resection. Radical surgery remains the standard treatment for this patient population.^424^ The feasibility of liver resection is clinically assessed based on patient tolerance, extent of the tumor, and residual liver function, often with systemic chemotherapy. This enables some patients to develop resectable tumors following the initial drug therapy.^425,426^ Surgical resection has been demonstrated to confer substantial survival benefits, with 5-year OS rates ranging from 20 to 50%.^427,428^ Conversely, the role of radiotherapy in the management of liver tumors is limited by concerns regarding local control and toxicity.^429^ SBRT is a precise and effective means of delivering high-dose radiation to liver metastasis. This approach effectively induces DNA damage and fragmentation, resulting in excellent local control with minimal side effects.^429^

It is estimated that over 50% of patients diagnosed with CRC will eventually develop metastasis, with the liver being the most common site of recurrence.^430^ In addition to downstaging tumors and converting unresectable to resectable cases,^431^ the use of 5-FU and leucovorin significantly enhances the median survival of patients with liver metastasis.^432^ The combination of irinotecan, fluorouracil, and leucovorin significantly prolongs PFS, improve response rates and extend OS.^433^ Moreover, the incorporation of oxaliplatin into fluoropyrimidine regimens markedly enhanced pathological complete response rates and reduced perioperative metastasis.^434^

Antiangiogenic agents are frequently used in targeted therapy for liver metastasis, often in combination with chemotherapy, to enhance efficacy. The incorporation of bevacizumab into first-line chemotherapy for metastatic CRC significantly increased the median PFS from 8.0 to 9.4 months.^435^ Furthermore, cetuximab, when combined with other agents, is well tolerated, reduces the risk of disease progression, and significantly increases the overall response rate (61% vs. 37%) compared to fluorouracil/leucovorin plus oxaliplatin.^436^

In addition to antiangiogenic therapies, immunotherapy has emerged as a promising treatment option. In patients with CRC and liver metastasis, responses to immune checkpoint blockade depend on the status of DNA microsatellite instability (MSI) or mismatch repair.^437,438^ In patients with high MSI (MSI-H) or deficient mismatch repair, nivolumab, a PD-1 inhibitor, achieved an objective response rate of 31.1%. This demonstrates durable responses and disease control and may represent a new treatment paradigm for these patients.^439^ The PD-L1 inhibitor atezolizumab, when used in combination with fluorouracil, leucovorin, oxaliplatin, irinotecan, and bevacizumab, has been demonstrated to be safe and effective in improving PFS.^440^

#### Targeting lung metastasis

The management of lung metastasis requires a multifaceted approach encompassing surgical, chemotherapeutic, radiotherapeutic, immunotherapeutic, and targeted therapeutic modalities, which are tailored to the stage and characteristics of the tumor.^189^ Specifically, surgical and radiotherapy procedures are primarily utilized to treat limited metastatic lesions, whereas chemotherapy, immunotherapy, and targeted therapy are more effective in addressing widespread systemic metastasis. However, chemotherapy is associated with considerable adverse effects, and certain agents, including paclitaxel^441^ and gemcitabine,^442^ may unintentionally contribute to the development of lung metastasis, necessitating caution among clinicians. Consequently, targeted therapy and immunotherapy, which offer substantial precision and reduced toxicity profiles, have emerged as promising avenues of research for the management of lung metastasis.

Aberrant gene expression is a key driver of tumor progression, treatment resistance, and lung metastasis.^443^ Notably, these dysregulated genes also represent novel therapeutic targets. Lung metastasis is a prevalent phenomenon in TNBC, and the response gene to complement 32 protein (RGCC) has been identified as a key driver of TNBC lung metastasis. This is achieved through the enhancement of PLK1 kinase activity, which drives AMPKα2 phosphorylation and subsequent downstream signaling. Notably, the combination of RGCC targeting with paclitaxel/carboplatin effectively inhibits TNBC lung metastasis in mice, indicating the potential of RGCC as a target for treatment strategies.^444^

Within the ECM, aberrant expression of collagen type X (COL10A1) in TNBC may activate the PI3K/AKT pathway, stimulating tumor-associated angiogenesis, and promoting TNBC growth and lung metastasis.^445,446^ Therefore, the downregulation of COL10A1 represents a potential therapeutic strategy. Tirapazamine, a pan-PI3K inhibitor, inhibits the development of lung metastasis in TNBC by targeting PI3K/Akt/mTOR complexes 1 and 2 (mTORC1/2), which reduces the expression of proteins associated with EMT.^447^ Similarly, plant-derived isoflavone ononin has been demonstrated to inhibit the expression of EMT markers and matrix metalloproteinases, thereby reversing the EMT process and attenuating the growth of TNBC tumors and the formation of lung metastasis.^448^

Targeting primary tumor cells can indirectly mitigate the progression of lung metastasis. To suppress nasopharyngeal carcinoma (NPC) lung metastasis, strategies include knockdown of aryl hydrocarbon receptor nuclear translocator-like 2 (ARNTL2) or targeting the PLUNC-NLRP3 inflammasome axis to promote NLRP3 ubiquitination and degradation, as well as inhibition of the AMOTL2/LATS/YAP pathway, which reduces NPC cell migration and invasion.^449,450^ In chondrosarcoma lung metastasis, nicotinamide phosphoribosyltransferase promotes lysyl oxidase (LOX) production via c-Src and Akt signaling, thereby enhancing LOX-dependent chondrosarcoma cell migration and invasion.^451^ In a study by Wang et al., inhibition of the c-MYC/Nrf2 pathway hindered the migration and invasion of CRC cells, thereby suppressing lung metastasis.^452^ In the context of osteosarcoma lung metastasis, the SDC4-TGF-β signaling feedback loop and Wnt/β-catenin pathway represent promising avenues for therapeutic intervention.^453,454^ Moreover, nerve growth factor (NGF) upregulates matrix metalloproteinase-2 (MMP-2) via the MEK/ERK pathway, thereby enhancing osteosarcoma cell migration and invasion.^455^ Larorectinib, a tropomyosin receptor kinase inhibitor, has been demonstrated to potently inhibit the effect of NGF on lung metastasis, thereby emerging as a potential therapeutic candidate for osteosarcoma lung metastasis.^455^

#### Targeting lymphatic metastasis

Lymph node metastasis (LNM) is a common feature of most solid malignancies and serves as a crucial basis for disease staging and prognosis. Furthermore, colonization of tumor cells within lymph nodes results in tumor immune tolerance, which facilitates distant metastasis and disease progression.^269^ Consequently, addressing LNM is of paramount significance for enhancing the prognosis of patients with cancer.

In the context of LNM, lymph node dissection has two principal functions. First, it prevents recurrence by eradicating tumor lesions in tumor-draining lymph nodes (tdlns). Second, it provides precise information for the staging, diagnosis, treatment, and prognostic prediction of patients with cancer.^265,456^ These effects positively influence tumor recurrence, metastasis, and survival. Radiotherapy, a crucial treatment modality for low-metastatic tumors, has markedly benefited patients with LNM. A large cohort study by Cavano et al. demonstrated that 90% of patients with LNM underwent SBRT in a safe and well-tolerated manner, resulting in high local control and survival rates.^457^ Among patients with nasopharyngeal carcinoma and parotid LNM, those who received intensity-modulated radiotherapy to the parotid lymph nodes exhibited superior survival outcomes compared to those with preserved parotid lymph nodes.^458^ Nevertheless, it is advisable that patients with cancer undergoing immunotherapy exercise caution when contemplating surgical or radiotherapeutic intervention, as lymph node resection has the potential to disrupt host immune structures and functions, thereby attenuating antitumor immune responses and potentially rendering immunotherapy ineffective.^459,460^ Furthermore, local treatments such as surgery and radiotherapy are frequently inadequate for addressing occult LNM. Therefore, it is imperative to investigate the potential of immunotherapeutic or targeted therapies for lymph nodes to manage LNM more effectively.

Lymphangiogenesis plays a pivotal role in facilitating tumor cell dissemination to the lymph nodes,^265^ rendering it a promising therapeutic strategy for LNM. The activated C kinase 1 receptor has been demonstrated to promote lymphangiogenesis in vivo through galectin-1-dependent mechanisms^461^ and activation of the glycolytic AKT/mTOR signaling pathway.^462^ This consequently facilitates the development of cervical cancer LNM. In addition, targeting enzymes involved in specific lipid metabolic pathways represents a promising avenue for the development of novel therapeutic strategies to treat cervical cancer LNM.^463^ In a recent study, Mei et al. demonstrated that 7-dehydrocholesterol reductase (DHCR7) plays a crucial role in promoting LNM by activating the KANK4/PI3K/AKT signaling pathway and enhancing VEGF-C secretion. These findings further validated that DHCR7 is a novel potential therapeutic target.^464^ Sterol O-acyltransferase 1 (SOAT1), which is overexpressed in gastric cancer, promotes lipid synthesis, induces lymphangiogenesis, and promotes gastric cancer LNM by upregulating VEGF-C expression. Therefore, SOAT1 could serve as a potential therapeutic target.^465^ Specific non-coding RNAs may emerge as potential targets to inhibit lymphangiogenesis, which could prove to be an effective strategy for the prevention and treatment of LNM. MicroRNAs such as miR-182-5p, miR-431-5p, and circular RNA NFIB1 and VESTAR, can inhibit tumor lymphangiogenesis by regulating VEGF expression.^466–469^

The inhibition of lymphatic metastasis can be achieved by targeting cancer cells directly. In head and neck squamous cell carcinoma, silencing of RING1 and YY1 binding protein expression^470^ and activation of the Wnt/β-catenin/Slug signaling axis^471^ contribute to cancer cell invasion and migration. In laryngeal cancer, silencing of HOXC6 inhibits EMT, viability, migration, and invasion of laryngeal cancer cells, thereby suppressing laryngeal cancer LNM.^472^ Li et al. demonstrated that MEOX1 promotes LNM in ovarian cancer by regulating multiple biological processes, including proliferation, EMT, lymphangiogenesis, and ECM remodeling. These findings suggest that MEOX1 may serve as a potential biomarker for ovarian cancer LNM diagnosis and treatment.^473^ In gastric cancer, RPRD1B promotes fatty acid uptake and synthesis, as well as LNM through activating the c-Jun/c-Fos/SREBP1 axis. This highlights RPRD1B as a potential target for gastric cancer LNM treatment.^474^

Therapeutic strategies targeting tumor metastasis demonstrate innovative pathways across multiple layers and dimensions. These advancements encompass a spectrum of approaches, ranging from precise attacks on metastatic cancer cells to intricate modulation of the complex TME, culminating in personalized treatments tailored to specific organ metastasis (Table 4). Each advance illustrates a profound understanding of tumor biology and a precise approach to treatment. With ongoing advancements in fundamental research and technology, these targeted therapies will continue to be refined and integrated, offering patients with cancer more efficacious, safer, and tailored treatment regimens. In conclusion, this progression promises to significantly extend patient survival and enhance QOL.

**Table 4:** Therapeutic strategies targeting organ-specific metastasis

Targeting organ-specific metastasis	Main therapeutic strategies	Key cellular participants	Signaling molecules/pathway/mechanisms	References
Bone	Monoclonal antibody	Osteoclast and osteoblast	RANKL, αv integrin, and VEGFR	^369–377^
	ADT	Prostate cancer cells	Androgen receptor	^381–385^
	Bisphosphonates	Osteoclast	Hydroxyphosphonite	^386,387^
	Radioisotopes	Cancer cells	Lead to ionization, generation of free radicals, and DNA strand breaks	^392,393^
Brain	Surgical intervention	/	/	^403,404^
	Targeted drug	Cancer cells	EGFR, ALK, HER-2, BRAF, MEK, and NRAS	^288,408–423^
Liver	Surgical intervention	/	/	^424–428^
	SBRT	Cancer cells	Induces DNA damage and fragmentation	^429^
	Chemotherapy	Cancer cells	Inhibit tumor growth or destroy tumors by chemical agents	^431–434^
	Anti-angiogenic therapy	Endotheliocyte	VEGF	^435,436^
	Immunotherapy	Immune cells	Enhance the capacity of immune system to accurately identify and eradicate cancer cells	^440^
Lung	Surgical intervention	/	/	^189^
	Chemotherapy	Cancer cells	Inhibit tumor growth or destroy tumors by chemical agents	^272,441,442^
	Targeted therapy	Cancer cells	PI3K/Akt/mTOR, NLRP3, AMOTL2/LATS/YAP, c-Src, Akt, c-MYC/Nrf2, SDC4-TGF-β, Wnt/β-catenin, and NGF	^447,449–455^
Lymph node	Surgical intervention	/	/	^265,456^
	Radiotherapy	Cancer cells	Induces DNA damage and fragmentation	^457,458^
	Targeted therapy	Cancer cells	AKT/mTOR, VEGF, RING1, YY1, Wnt/β-catenin/Slug, MEOX1, RPRD1B, and c-Jun/c-Fos/SREBP1	^462,464,465,470–474^

## Emerging therapeutic technologies

The accelerated advancement of science and technology has facilitated the application of numerous emerging technologies in tumor metastasis research and treatment. These innovative methodologies enhance therapeutic efficacy, minimize side effects, and offer personalized and precise solutions to the complexities of metastatic cancers (Table 5). This progress brings new hope and potential for the treatment of tumor metastasis.

**Table 5:** Advantages and disadvantages of emerging therapeutic technologies for tumor metastasis treatment

Emerging technology	Advantages	Disadvantages	Potential application	Examples of applications
Nanotechnology and nanomaterials	Precise targeting potential; regulated release potential; high loading and structural tunability;	Toxicity and safety; stability and long-term effects; difficult to control delivery and release; manufacturing and scale-up challenges;	Targeted drug delivery systems	Enzyme/pH dual stimuli-responsive nanoplatform co-deliver disulfiram and doxorubicin for treating breast cancer lung metastasis.^657^
			Enhanced imaging performance	Nanocomposites based on Mn_3_O_4_ achieved accurate monitoring and diagnosis of gastric cancer metastasis by supporting MRI/fluorescence imaging (FLI) dual-modality imaging.^475^ Near-infrared dye-sensitized upconversion nanoparticles for long-term monitoring of tumor metastasis.^658^
			Targeting TME	Self-delivery micellar nanoparticles prevent PMN formation.^659^ Micellar nanoparticles can improve the inflammatory and immunosuppressive microenvironment of the lung and tumor sites.^660^ Radioactive nano-oxygen generator can enhance the infiltration of CTLs and reduce tumor cell proliferation.^661^
			Interfering with the process of tumor metastasis	Photothermal-controlled NO-releasing Nanogels reversed EMT.^662^ Cryoprotective isoliquiritigenin-zein phosphatidylcholine nanoparticles suppressed breast cancer cell proliferation, colony formation, and motility.^663^ Sponge-like nano-system decreased the permeability of pulmonary vessels and inhibited the implantation of CTCs.^664^ Glycopolymer-grafted nanoparticles can weak the adhesion between tumor cells and activated platelets for inhibiting tumor metastasis.^665^
			Theranostic System	Semiconducting Polymer Nanoparticles were used for NIR-II fluorescence imaging and PTT.^479^ A novel nanodiagnosis-treatment agent (Ag@CuS-TPP@HA) accomplishes targeted the NIR-II PA imaging of tumor tissue and leverages ROS/photothermal therapy to enhance immune checkpoint blockade.^666^
			Immuno-enhancing therapy	The engineered nanoagonist facilitated the maturation of dendritic cells and infiltration of cytotoxic T lymphocytes for long-term anti-tumor immunity to suppress tumor metastasis.^667^ A novel core-shell integrated nano platform enhanced cancer immunotherapy by targeting TAMs and repolarizing TAMs.^668^
			Combined with other therapies	Hybrid semiconducting polymer nanoparticles (SPN_H_) diagnose and treat breast cancer by combining PDT, PTT and SDT.^487^ A novel multifunctional biomimetic nanovaccine achieves highly efficient chemodynamic immunotherapy.^528^ Metal-organic framework-mediated multifunctional nanoparticles can combine chemotherapy with PTT for colorectal cancer treatment.^669^ A nano ultrasound contrast agent (arsenic trioxide (ATO)/PFH NPs@Au-cRGD) inhibits lung metastasis by enhancing Chemo-photothermal Therapy and anti-programmed death ligand 1 immunotherapy.^670^
Gene editing technology	Correcting genome mutations; studying phenotypic effects of genes; high specificity; versatility; targetability of the desired tissue or organ; precision medicine based on individual’s tumor genetics;	Insufficient safe delivery; poor gene editing efficiency; biosafety; off-target effect; ethical issues; unknown long-term outcomes;	Editing Cancer Cells	Co-delivery of Cas9 mRNA and gRNA reduced the migration and invasion capacity of cancer cells by editing the LGMN gene.^495^ PIM3 knockout impaired the formation of lung metastasis.^496^
			Screening for potential therapeutic targets	High MEST expression promoted tumor cell invasion and metastasis.^671^ LncRNA LINC00982 is a novel regulator in cancer metastasis and drug resistance of colorectal cancer by expressing a protein PRDM16-DT.^672^ Dickkopf-1 (DKK1) promotes the growth and metastatic dissemination of mCRPC.^673^ FOXQ1 promotes tumor progression when losing normal p53 function.^674^
			Enhancing immunotherapy	Editing ex vivo T cells from patients with CRISPR/Cas9 system to help improve cancer immunotherapy.^675^
			Interference with the metastasis process	Stromal-specific knockout (cKO) of Yap1 impedes PMN formation and metastatic progression of ovarian cancer by suppressing YAP1/GROα/CXCRs signaling cascade.^676^ Reducing extracellular matrix stiffness by CRISPR/Cas gene editing.^677^
			Combining other technologies	Calcium phosphate-based nanoparticle delivery genome editing system for treating CRPC.^678^ A CRISPR/Cas9-based genome-editing nanomedicine inhibits tumor growth and metastasis.^679^
Cancer vaccine	High specificity; personalized immunotherapy; low cost and safety; preventive therapeutic effect;	Intrinsic tumor cell resistance; potential off-target risks; lacking of effective immune response; local or systemic immunosuppressive mechanism;	Activating immune cells	Stimuli-responsive mRNA Vaccines amplified the magnitude and function of antigen-specific CD8 + T cells for systemic antitumor efficacy.^512^ A novel peptide-based tumor nanovaccine elicits robust tumor antigen-specific CD8 + T cell response.^511^
			Enhancing antigen presentation	Capturing and transporting tumor antigens to enhance cancer immunotherapy by promoting the efficient presentation of tumor antigens.^680^
			Modulating immunosuppression	Targeting TAMs: A dual-targeting nanovaccine reduces in situ and abscopal tumor growth by boosting T cells and repolarizing M2-like TAMs.^530^ Targeting MDSC: DNA vaccine combines with PI3Kγ inhibition to target MDSC for strengthening anti-tumor response.^513^ Targeting neutrophils: Sialic acid-modified liposomes combine with scaffold-based vaccines to inhibit postoperative tumor recurrence and progression by overcoming neutrophil-induced postoperative immunosuppression.^681^
			Prevention of tumor metastasis	Mucosal tumor vaccine prevents and treats pulmonary metastases.^510^
			Visual detection	The MRI-trackable nanovaccine provides imaging-guided immunotherapy and real-time monitoring of the immunization process.^525^
			Personalized treatment	Toll-like receptor 7/8 agonist-epitope conjugate for personalized immunotherapy.^682^ Tumor vaccine based on Hybrid Ginseng-derived Extracellular Vesicles-Like Particles with the membrane of the resected autologous tumors suppresses tumor recurrence and metastasis.^531^
			Theranostic System	Biomimetic dual-target theranostic nanovaccine achieves MR imaging and combination chemo-, chemodynamic-, and immune therapy of orthotopic glioma.^683^
			Combination therapy	Laser-activatable in situ vaccine enhances anti-tumor immunity by targeting the multiple key steps in the cancer-immunity cycle and combining PTT, PDT and anti-programmed death ligand 1 antibody.^684^
Artificial intelligence (AI)	Standardization and quality control; improving efficiency and accuracy; predictive analytics; personalized treatment; reducing workload; unbiased; reviewing tool;	Legal and ethical considerations; privacy and security issues; heterogeneity among datasets; risk of overfitting; poor interpretability; poor generalizability; lacking of external validation; cost and implementation challenges;	Precision medicine	Using AI and MRI signatures to predict non-invasive molecular subgroups in medulloblastoma.^542^
			Tumor Origin Assessment	Predicting origins for cancers of unknown primary by using AI-based pathology.^685^ Identifying malignancy and predicting tumor origin in both hydrothorax and ascites by using cytology-based deep learning.^686^
			Predicting tumor metastasis	Based AI-trained thyroid ultrasound predicts cervical lymph node metastasis in patients with papillary thyroid carcinoma.^687^ Based on AI-guided histopathology predicting brain metastasis in patients with lung cancer.^688^
			Cancer Risk Assessment	An AI-based risk model for risk assessment in breast cancer.^541^ Predicting the risk of liver metastasis in patients with Gastrointestinal stromal tumors by constructing machine learning algorithms.^689^
			Predicting treatment response	Longitudinal ultrasound-based AI model predicts axillary lymph node response to neoadjuvant chemotherapy in breast cancer.^690^
			Recognition and diagnosis of tumor metastases	Identifying lymph node and distant metastases on whole-slide images of breast cancer based on the AI platform.^691^ Diagnosing lymph node metastases on whole-slide images of bladder cancer based on AI model.^692^ Diagnosing upper gastrointestinal cancers by using endoscopy imaging based on the gastrointestinal AI diagnostic system.^693^ Evaluating the invasion depth of early gastric cancer through AI-based endoscopic ultrasonography diagnostic system.^694^
			Predicting patient prognosis	Predicting overall survival in lung cancer with missing values based on AI model.^695^ Predicting 1-year survival after palliative radiotherapy for bone metastasis based on machine learning model.^696^ Predicting the prognosis of patients with TNBC by using AI-based analysis.^697^
			Omics data analysis	Predicting the origin of cancer of unknown primary by using DNA methylation datasets.^698^
			Exploring and developing biomarkers	POLD1 as a novel biomarker related to prostate cancer metastasis.^699^ Altered PCK1 and LPL expression as key in breast cancer metastasis recurrence.^700^
Bioinformatics	Analysis and clinical interpretation of High-throughput tumor molecular profiles; trace the origin of particular cell lineages; multi-omics studies; exploring precision medicine applications; availability and ease, access, affordability; improving research efficiency;	Limited data availability; algorithmic bias; privacy and regulations issues; interpretability and transportability; electronic annotations are not entirely accurate; lack of analytical tools and trained personnel;	Screening for potential therapeutic targets	DNA topoisomerase II alpha (TOP2A) might be a potential therapeutic target for anti-metastatic therapy.^701^ ERBB3 was identified as a key gene with therapeutic implications in thyroid carcinoma.^702^
			Exploring and developing cancer biomarkers	Anoikis-related genes FASN and RAC3 may become new potential biomarkers for the diagnosis and treatment of bladder cancer.^559^ The circRNF216 is a potential biomarker for diagnosing and treating colorectal cancer.^703^
			Drug development	Dihydroartemisinin may inhibit tumor metastasis by inhibiting angiogenic pathways in melanoma cells.^704^ Ruangan Lidan decoction (RLD) may inhibit liver cancer growth and metastasis.^705^ Jinfukang (JFK) promotes the infiltration of CD8 + T and NK cells in tumor tissues to reduce the burden of lung cancer metastasis.^706^
			Identifying metastasis-related genes and pathways	Endosomal protein DENND10 is associated with cell spreading, migration, invasion, and metastatic potential.^707^ Long non-coding RNA LINC00909 promotes cancer stemness and metastasis by inhibiting SMAD4 expression.^708^ RING Finger Protein 115 (RNF115) triggers cell proliferation, EMT, and tumor metastasis by ubiquitinating and degrading CDK10 in thyroid carcinoma.^709^ Suppressor of cytokine signaling 2 (SOCS2) may inhibit the migration and invasion of hepatoblastoma cells by inhibiting the JAK2/STAT5 signaling pathway.^710^
			Predicting prognosis	Predicting the prognosis of glioma based on the SNARE proteins.^711^
			Personalized treatment	Predicting metastatic risk and organotropism and guiding clinical stratification for optimal treatment selection.^565^

*PMN* pre-metastatic niche, *EMT* epithelial-mesenchymal transition, *CTC* circulating tumor cell, *CTLs* cytotoxic T cells, *TAMs* tumor-associated macrophages, *MDSC* myeloid-derived suppressor cell, *CRPC* castration-resistant prostate cancer, *PDT* photodynamic therapy, *PTT* photothermal therapy, *SDT* sonodynamic therapy, *mCRPC* metastatic castration-resistant prostate cancer, *TNBC* Triple-negative breast cancer, *PI3Kγ* phosphoinositide-3-kinase γ

### Nanotechnology and nanomaterials

In recent years, the application of nanotechnology in medicine, particularly in treating tumor metastasis, has attracted considerable attention. Nanomaterials, with their distinctive properties and capabilities, present novel avenues for the early detection, diagnosis, and treatment of tumor metastasis. Nanomaterials can be used as contrast agents to enhance the resolution and sensitivity of imaging techniques, thereby improving the accuracy of tumor metastasis detection.^475,476^ Nanomaterial-based targeted drug delivery systems use surface-modified nanoparticles to selectively bind to tumor cells, facilitating precise drug release and enhancing efficacy.^477,478^ This approach addresses the limitations of traditional treatments, including their poor selectivity and high toxicity. Nanomaterials have the potential to construct an integrated diagnostic and therapeutic platform, thereby synchronizing disease diagnosis and treatment, and improving medical efficiency and therapeutic efficacy.^479,480^ The application of nanotechnology has markedly advanced research on the complex interplay between TME, metabolism, and therapeutic interventions, thereby facilitating the development of innovative therapeutic strategies. Some nanomaterials can release antitumor drugs in response to specific conditions such as pH, temperature, and enzyme activity within the TME.^481–483^ Moreover, nanomaterials have the potential to impede tumor cell invasion and metastasis by influencing cell adhesion and matrix remodeling, among other mechanisms.^484–486^ Furthermore, nanomaterials can be combined with therapeutic strategies such as immunotherapy and radiotherapy to facilitate the effectiveness of combined therapy.^480,487–489^ Novel nanomaterials and techniques have led to the expectation that nanotechnology will facilitate transformative advances in cancer therapy. Nevertheless, it is imperative to underscore the biosafety, long-term effects, and potential side effects of nanomaterials.^490^

### Gene-editing technologies

Gene-editing technology, particularly CRISPR/Cas9, has significantly advanced life science research, offering immense promise for advancing precision tumor medicine.^491–493^ In the context of tumor metastasis, gene editing has the potential to inhibit tumor growth and metastasis by knocking out or repairing oncogenes and mutated genes.^494–496^ Furthermore, gene editing can identify and characterize genes associated with tumor metastasis, which may offer potential therapeutic targets for antitumor metastasis treatments.^497,498^ Moreover, gene editing facilitates the identification of genes responsible for tumor drug resistance, thereby offering potential targets for addressing this significant challenge in cancer therapy.^499,500^ Furthermore, it can modify the expression of cells or molecules within the TME, thereby inhibiting tumor invasion and metastasis.^501–503^ Beyond direct gene editing, the application of nano-biomaterials and stimulus-responsive delivery strategies offers innovative approaches to enhance gene-editing efficiency and precisely control release,^504,505^ thereby mitigating toxicity and side effects. Nevertheless, gene-editing technologies still face significant challenges, including ensuring specificity and safety, avoiding off-target effects, and addressing ethical and legal concerns.^506,507^

### Cancer vaccines

Cancer vaccines, which stimulate effective antitumor immune responses by activating the immune system and immune cells, have the potential to inhibit tumor growth and metastasis.^508^ These vaccines can be classified into two main categories: prophylactic and therapeutic vaccines. In addition, they can be further distinguished based on their composition or carrier, which includes whole-cell tumor vaccines,^509^ tumor antigen vaccines,^510^ peptide vaccines,^511^ RNA vaccines,^512^ DNA vaccines,^513^ dendritic cell vaccines,^514,515^ nanovaccines,^516^ in situ vaccines,^517,518^ and bacterial nanovaccines.^519^ The integration of nanotechnology,^520,521^ biomaterials,^522^ and gene-editing technology,^523^ among other techniques, has the potential to further reduce off-target effects and enhance antitumor immune responses, thereby expanding the scope of treatment of tumor metastasis. Cancer vaccines can facilitate the aggregation of antigens in lymph nodes, thereby enhancing antigen presentation and initiating effective antitumor immune responses.^524–527^ Moreover, cancer vaccines can directly activate immune cells, thereby eliciting antitumor immunity.^528^ Furthermore, these vaccines can augment the efficacy of tumor cell destruction by modulating immunosuppressive mechanisms.^513,529,530^ The development of vaccines based on specific tumor cell antigens from individual patients allows for a personalized treatment approach.^509,531^ Moreover, cancer vaccines with visualization and real-time monitoring are rapidly evolving, facilitating a deeper understanding of the mechanisms and principles of vaccines in vivo. This enhances the efficacy of the cancer vaccines.^525,532^ Nevertheless, cancer vaccines face several challenges, including immune evasion of tumor cells, optimization of vaccine design, and immunosuppression of the TME.

### Artificial intelligence

Artificial intelligence (AI) technologies, including machine learning and artificial neural networks, can be utilized for tumor risk stratification, metastasis detection, and treatment response. These technologies are undergoing rapid development for the treatment of tumor metastasis.^533–535^ AI has the potential to markedly enhance the diagnostic accuracy and efficiency of clinicians through its assistance in medical image analysis, thereby reducing diagnostic time and minimizing the risk of missed diagnosis.^536,537^ Moreover, AI can analyze vast biological data to predict drug targets, thereby accelerating the development of new drugs and reducing associated costs.^538,539^ Furthermore, AI can scrutinize patients’ genomic and clinical data, facilitating the identification of gene mutations, assessment of cancer risk, drug sensitivity stratification, treatment response prediction, and prognostic modeling, thereby enabling precision medicine.^540–546^ The advent of large language models, such as ChatGPT, is anticipated to facilitate the application of generative AI (GAI) in several critical areas, including clinical document writing and data management,^547–550^ as well as clinical decision assistance^551,552^ and personal healthcare.^553^ Nevertheless, several challenges remain to be addressed, including the interpretability of AI, data bias, and protection of privacy.^554^

### Bioinformatics

Bioinformatics is an interdisciplinary field that draws on knowledge from biology, computer science, and mathematics to address complex biological issues.^555^ It plays a pivotal role in research on tumor metastasis. Bioinformatics can analyze and interpret a vast quantity of biological data, which serves as the foundation for uncovering the biological characteristics, metastatic mechanisms, and regulatory mechanisms of tumor metastasis.^556–558^ Furthermore, bioinformatics facilitates the discovery and identification of potential biomarkers for the early diagnosis, treatment monitoring, and prognosis assessment of tumor metastasis.^559,560^ By examining the molecular characteristics of cancer cells, bioinformatics can identify potential new therapeutic targets that can inform the design and optimization of novel anticancer drugs.^561–564^ Moreover, bioinformatics can integrate patients’ genomic, transcriptomic, and proteomic data with medical imaging and treatment response data.^565^ This integrated approach provides a valuable reference for formulating personalized treatment plans. However, the application of bioinformatics also encounters challenges, including data quality and availability, the accurate annotation of information, fairness, and ethical considerations.^566–568^

## Challenges of studying cancer metastasis

### Knowledge gaps in understanding tumor metastasis

The dissemination of metastasis raises several significant questions, particularly regarding the timing of this process. It is postulated that dissemination may commence in the initial stages of tumor progression, with the asymptomatic establishment of PMN occurring at this stage. This early and often undetectable phase presents challenges for clinical diagnosis and early intervention, thus complicating studies on PMN in human patient samples. Consequently, it is difficult to understand how advancements in our knowledge of PMN could translate into clinical benefits for cancer treatment.

Recent genomic analyses and lineage-tracing experiments have revealed that numerous metastasis evolve through a metastasis-to-metastasis route.^68,569–573^ In bone metastasis, Zhang et al. discovered that tumors metastasizing to the bone enhance their stemness and metastatic capabilities via epigenetic modifications.^574^ However, comprehensive studies comparing transcriptomic, epigenetic, and genetic alterations between tumor cells from primary and secondary metastasis are lacking. This limitation arises because current methodologies cannot reliably differentiate the cellular origin of CTCs, whether from surgical operations during cell line injections, primary metastasis, or further advanced metastasis.

The phenomenon of metastasis organ tropism, coupled with the observation of metastasis-to-metastasis, indicates that tumor progression occurs with host tissue evolution. A single type of cancer can metastasize to various tissues, whereas different cancers can metastasize to the same organ. However, there is a paucity of comprehensive studies comparing metastatic ecosystems across various tissues from the same cancer type or different cancer types that metastasize to the same organ. Some research groups have identified this knowledge gap and have focused on delineating tumor-host co-evolution. For example, Xu et al. comprehensively analyzed lung cancer metastatic TME in various organs, including the bone, liver, and brain.^575^ Similarly, Liu et al. examined the diverse immune archetypes of bone metastasis colonized by different cancer types.^576^ Both studies used single-cell sequencing, underscoring the need for more extensive high-throughput analyses. Such profiling could enhance our understanding of cell-cell signaling and immune-tumor interactions within the metastatic TME, elucidate organ tropism mechanisms, and inform potential treatments for metastasis. These studies are vital for developing early interventions to prevent metastasis, identifying biomarkers for prognosis, and tailoring personalized therapies to improve patient outcomes. The paucity of high-throughput screening of metastatic TMEs is attributable to several factors. The establishment of organ-specific spontaneous metastasis in mouse models is challenging because of the lack of ideal models that eliminate the influence of surgical interventions and excessive immune response to cell line tags. Moreover, in vivo metastatic models are inherently difficult to control. Obtaining matched primary and metastatic samples from patients is a significant challenge, particularly given the invasive nature of the procedures involved and the necessity for coordination across diverse healthcare systems. Furthermore, patients who undergo surgical procedures often introduce substantial batch effects, which can complicate data analysis and interpretation.

Single-cell sequencing techniques have been widely used in studies on the TME. However, tumor metastasis is dynamic, with significant cellular and molecular kinetics constantly evolving. Profiling samples from a specific time point provides only a partial representation of the entire metastatic process. Despite the availability of sophisticated analytical tools, such as monocle 3^577–580^ and RNA velocity,^581,582^ which facilitate the estimation of cellular state transitions at high resolution across experimental conditions, these techniques remain inadequate for fully capturing the actual lineage trajectories. The accuracy of trajectory inference is challenging to evaluate, given the extensive reprogramming events that occur in the TME. Furthermore, data preprocessing of single-cell datasets has the potential to eliminate rare cell populations, which could result in analyses skewed by dominant cell populations.

### Obstacles of cancer metastasis in clinical trials

Clinical trials are an indispensable component in the process of acquiring high-quality therapeutic evidence and assessing the efficacy and safety of unlisted drugs. They play a pivotal role in accelerating the development of antitumor metastatic drugs, thereby meeting the needs of patients with cancer.^583^ Nevertheless, clinical trials are confronted with a multitude of challenges that impede the acquisition of superior medical evidence and profoundly affect the diagnosis and treatment of tumor metastasis.

#### Experimental design

Randomized controlled trials remain the gold standard for evaluating treatment regimens and efficacy in tumor metastasis, providing robust evidence for developing clinical treatment protocols.^584–586^ Organ-specific tumor treatment strategies have yielded notable therapeutic outcomes in numerous tumor types. This success prompted the design of numerous clinical trials based on primary tumors to achieve optimal efficacy. However, metastatic tumors frequently exhibit greater complexity than their primary counterparts, rendering primary tumor-based treatment regimens ineffective in the context of metastatic disease.^380,587^ Therapeutic regimens specifically tailored to the characteristics of metastasis have demonstrated favorable outcomes.^387^ One such example is the use of bone modifiers to treat bone metastasis. Moreover, in light of the difficulties encountered when attempting to achieve optimal therapeutic effects with a single-treatment approach, combination therapy has increasingly become the primary focus of tumor metastasis treatment.^588^ Therefore, well-designed clinical trials targeting the distinctive characteristics of tumor metastasis should be conducted to enhance efficacy in patients with tumor metastasis and obtain high-quality evidence-based medical evidence.

#### Tumor heterogeneity

The heterogeneous nature of tumors is a consequence of the selective pressure exerted by the TME. Primary tumor cells and distant metastatic cells exhibit differential metabolic processes, immune responses, growth rates, and other characteristics that collectively contribute to tumor heterogeneity.^589^ This phenomenon is a crucial indicator of cancer progression.^590,591^ Tumor heterogeneity enhances cancer cell invasiveness and promotes drug resistance, which is a significant factor in the development of drug resistance.^589,592–594^ Targeted therapy represents a strategy to address drug resistance in cancer cells; however, it is also susceptible to drug resistance.^379,380,587,595,596^ The results of clinical trials have revealed notable discrepancies in the efficacy of treatment regimens when administered to subgroups of patients with disparate genetic profiles.^585,593^ This indicates that a significant reason for the failure of targeted therapy for tumor metastasis may be attributed to the fact that targeted therapy is based on the distinctive gene expression of the primary tumor rather than metastatic cancer cells.^597,598^ Consequently, when conducting clinical trials about the treatment of tumor metastasis, it is imperative that researchers consider tumor heterogeneity and utilize biomarker-based methodologies to identify patient subgroups that respond to targeted pharmaceuticals, thereby optimizing the therapeutic outcomes.^599^

#### Non-experimental factors

In the context of clinical trials, investigators must consider a multitude of non-experimental factors, including the composition of the research team, availability of patient resources, financial constraints, accessibility of equipment, and prevailing ethical regulations. If these factors are not effectively managed, they can impede the standard conduct of clinical trials. The availability of patient resources is a prerequisite for clinical research. The successful implementation of clinical trials is contingent upon the enrollment of eligible patients and the attainment of a specified sample size and scale.^600–603^ Moreover, the considerable financial costs associated with conducting these trials are a significant factor.^604^ A substantial number of clinical trials require costly infrastructure to facilitate their studies. Consequently, the applicability of their conclusions is contingent on cost-effectiveness.^605^ It is becoming increasingly common for different institutions and countries to collaborate to reduce costs and accelerate patient recruitment.^585^ Furthermore, the lack of specialized personnel, inadequate equipment availability, prolonged turnaround times, and discrepancies in ethical regulations can impede the appropriate conduct of clinical trials.^601,606^ Therefore, investigators must consider and manage these non-experimental factors during the design and execution of clinical trials to promote the normal, safe, and orderly implementation of clinical trials.

## Conclusions and future direction

In recent decades, significant advancements have been made in tumor metastasis research, particularly in malignant diseases. Researchers have elucidated intricate mechanisms and emphasized the pivotal role of the TME as a crucial regulator in this dynamic process. From the intricate modulation of cellular signaling pathways to the complex interplay within the ECM and the intricate cellular and molecular dynamics across various host organs, all have been identified as key factors influencing tumor metastasis. The particular characteristics of multiorgan metastasis, including those to the lungs, liver, brain, and bones, along with their underlying molecular mechanisms, have further enhanced our comprehensive understanding of the landscape of tumor metastasis. Recent clinical trials have not only deepened our understanding of the intricate relationships between tumor metastasis, primary tumor characteristics, treatment responses, and patient outcomes but have also fueled the development and application of diverse strategies targeting specific signaling pathways and innovative therapeutic targets. These efforts have enhanced the efficacy of therapeutic interventions and have the potential to extend patient survival and improve QOL. Further research is required to gain a deeper understanding of the mechanisms by which the TME influences tumor metastasis. A comprehensive examination of the intricate interactions between tumors and various host tissues, organs, immune cells, vascular networks, and other microenvironmental components is required to identify novel therapeutic targets and intervention strategies. Concurrently, we seek to gain molecular-level insights into the entire process of tumor metastasis by applying advanced bioinformatics, genomics, and proteomics technologies, thereby enabling more precise monitoring and intervention in disease progression. As data accumulates and technology advances at an unprecedented pace, the vision of personalized medicine is becoming increasingly realized. The implementation of customized treatment regimens based on individual genetic profiles, protein expression patterns, and other biomarkers will facilitate a more precise match with patients’ therapeutic needs, thereby enhancing treatment efficacy while minimizing adverse effects. This paradigm shift in treatment modalities undoubtedly offers hope to patients with cancer, heralding a new era of tumor therapy. In conclusion, a comprehensive and intensive study of tumor metastasis has deepened our understanding of its complex biological processes and laid a solid foundation for the development of more effective and individualized treatment strategies. These endeavors have the potential to markedly enhance patients’ survival rates and QOL, thereby contributing to the global initiative to surmount this formidable health challenge.
